# A Universal Trend among Proteomes Indicates an Oily Last Common Ancestor

**DOI:** 10.1371/journal.pcbi.1002839

**Published:** 2012-12-27

**Authors:** Ranjan V. Mannige, Charles L. Brooks, Eugene I. Shakhnovich

**Affiliations:** 1 Department of Chemistry and Chemical Biology, Harvard University, Cambridge, Massachusetts, United States of America; 2 Department of Chemistry and Biophysics Program, University of Michigan at Ann Arbor, Ann Arbor, Michigan, United States of America; 3 Department of Molecular Biology, The Scripps Research Institute, La Jolla, California, United States of America; 4 Center for Theoretical Biological Physics, University of California San Diego, La Jolla, California, United States of America; UT Southwestern Medical Center at Dallas, United States of America

## Abstract

Despite progresses in ancestral protein sequence reconstruction, much needs to be unraveled about the nature of the putative last common ancestral proteome that served as the prototype of all extant lifeforms. Here, we present data that indicate a steady decline (oil escape) in proteome hydrophobicity over species evolvedness (node number) evident in 272 diverse proteomes, which indicates a highly hydrophobic (oily) last common ancestor (LCA). This trend, obtained from simple considerations (free from sequence reconstruction methods), was corroborated by regression studies within homologous and orthologous protein clusters as well as phylogenetic estimates of the ancestral oil content. While indicating an inherent irreversibility in molecular evolution, oil escape also serves as a rare and universal reaction-coordinate for evolution (reinforcing Darwin's principle of Common Descent), and may prove important in matters such as (i) explaining the emergence of intrinsically disordered proteins, (ii) developing composition- and speciation-based “global” molecular clocks, and (iii) improving the statistical methods for ancestral sequence reconstruction.

## Author Summary

Although of importance to both evolution and protein design, the manner in which the first proteome came to be, and the actual features of the earliest ancestral proteomes are both unknown. Through the analysis of diverse proteomes, we provide glimpses into the composition of the last common ancestor (LUCA) of all lifeforms, which indicate that the earliest/last common ancestor had a proteome that was highly hydrophobic/oily. Notably, the evidence presented (a) indicates that proteomes of all species ranging from bacteria to mammals appear to adhere to the same universal constraint (“oil escape”) set into motion by the last common ancestor more than 3.5 billion years ago, (b) indicates the presence of a previously untapped global (composition-level) molecular clock, and (c) strengthens the non-equilibrium/directional view of amino acid substitutions that challenges central dogmas regarding reversibility in molecular evolution.

## Introduction

What did the proteome of the first successful lifeform look like? This question is critical to the understanding of how life as we know it first began on earth. Despite the progresses in ancestral sequence reconstruction methods [1]–[3], predicting the proteome of the Last Common Ancestor (LCA) from today's proteomes is impeded by the inevitable accumulated errors implicit in any extrapolation method [4]. Also, while interesting predictions regarding ancestral genome behavior can be made [5], [6], the sequence reconstruction methods utilized to make those predictions are fraught with precarious (or even potentially “flawed”) presuppositions [7]–[11]. Here we re-address the question of what the composition of the LCA may have been, and provide an answer sourced from simple considerations that our LCA might have had a highly hydrophobic (oily) proteome, which has slowly been equilibrating over evolutionary time. The results are obtained independently of sequence reconstruction methods and previously published statistical techniques [1]–[3], [5], [6], which provides a method-independent snapshot into the molecular nature of the last common ancestor.

**Figure 1:**
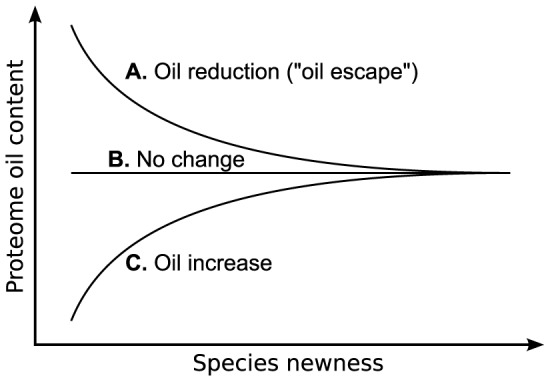
Three scenarios exist for the monotonic drift or evolution of oil content over time, predicated upon whether the last common ancestor's oil content is higher (A), equal (B) or lower (C) than the oil content that is expected from sequence entropy considerations.

It is important to first introduce the notion that proteome *compositions* equilibrate at “glacial” speeds (over billions of years; Section S1.1 in Text S1, and discussed below), which will be important in extrapolating trends obtained from present proteomes to properties of the LCA's proteome. While mutations accumulate within a proteome at a relatively steady rate (to the order of about 1 substitution per billion years per nucleotide site [12]), the oil composition of that proteome–described as the cumulative percent composition within the sequence (%FILV) of the four most hydrophobic residues as per the Kyte-Doolittle scale [Phenylalanine (F), Isoleucine (I), Leucine (L) and Valine (V)]–is expected to change at a much slower rate due to reasons such as the proteome's massive size (Section S1.1 in Text S1) and the biochemical impediments associated with drastically changing a protein's composition (Section S1.2 in Text S1). Given this expected glacial drift/equilibration in proteome composition, in accordance with previous discussions [5], one can expect one of three general trends in the change of proteome oil content over even large evolutionary time (negative, neutral or positive correlations which is dependent on the LCA's original proteome oil content; Figure 1).

Also important to tracking changes in oil content over evolutionary time is the finding that, although all species have existed in *some* form for equal amounts of physical time (an expected outcome of common descent), their genomes are not equally *deviated* from the last common ancestor (Sections S1.3 and S1.4 in Text S1); the number of non-synonymous nucleotide substitutions accumulated since emerging from the LCA appears to be proportional to the number of speciation events that the species has encountered sans phylogenetic errors [13]–[15]. What this indicates is that, while a component of molecular evolution is due to the “constantly ticking” molecular clock caused by neutral or nearly neutral mutations [16]–[19], another component of genetic deviation from the LCA may be attributed to substitutions associated with speciation events (roughly proportional to species node number in the tree of life; see Methods), even though the exact extent and mechanism of substitutions in this regime is not known [15]. Importantly, it is especially expected that substitutions causing changes in oil content, due to being quite the opposite of neutral in fitness effects (Section S1.2 in Text S1), are expected to dominantly occur not during neutral drift but during the non-neutral component of molecular evolution, i.e., in events such as speciation (Section S1.4 in Text S1).

In this report, we use species node number as a measure of evolutionary age or “deviatedness” from the LCA (also roughly proportional to organismal complexity) to study the changes in proteome and protein composition over evolutionary time. While this study may be susceptible to the various expected *local* inaccuracies involved in building the tree of life (ToL), the global trend–that lower node-number organisms are “older” genomes with less genomic deviation from the LCA–is a notion that is acceptable, and such a precedence has been set [13]–[15], [20]. Additionally, given our interest in finding potential *low*-resolution or global trends over evolutionary time, the utility of node number is uniquely warranted.

## Results

### A common trend (“oil escape”) observed across all proteomes

**Figure 2:**
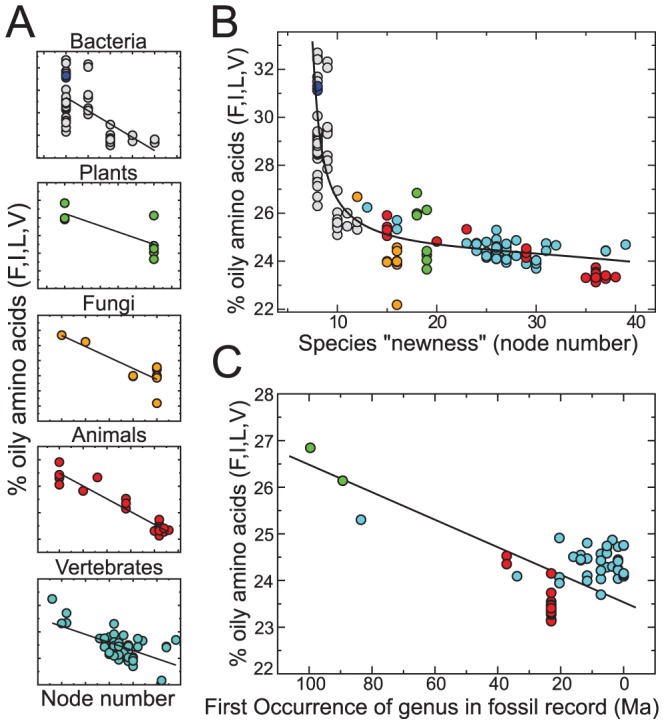
Proteome oil content reduces over “evolutionary time”. Proteome databases, both individually (**A**; detailed in Figure S1 in Text S1) and cumulatively (**B**; number of data points, 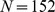), indicate a steady reduction in proteome oil content (%FILV) over evolutionary time (organism node number), with a Spearman rank correlation coefficient 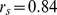 and probability 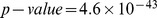. Another metric for evolutionary age (paleobiology's “First Occurrence” records; **C**) reiterates this trend (Spearman 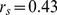, 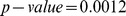, 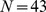; improved to 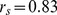, 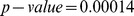 when binned per the abscissa), indicating the existence of a “super-oily” predecessor to all that exists. The three archeal proteomes obtained from Ensembl Bacteria are denoted by the blue-filled circles in (**A**, top) and (**B**).

We obtained and studied all of the proteomes available within the Ensembl genome databases (272 diverse proteomes belonging to 152 distinct species sourced from all domains of life; listed in Section S5 in Text S1) for a relationship between a species node number and its proteome oil content. Remarkably, the proteomes displayed a striking, universal relationship between the proteome oil content (%FILV), and the species node number in both individual Ensembl databases (Figure 2A; annotated axes in each panel are different and elaborated in Figure S1A in Text S1) and the merged data (Figure 2B), which is unexpected given the high diversity of the proteomes studied and the coarse nature of the ToL. Other metrics for oil content (hydrophobicity scales) showed similar results (Figure S2 in Text S1); however, %FILV provided the strictest trend and so is kept as the main metric henceforth.

Given the highly diverse nature of the organisms in our collection, environmental variables are not expected to contribute strongly to this universal trend (also discussed later in Other Trends); Oil escape is also reasserted (albeit loosely) by paleobiological records, where the relationship between proteome oil content and genus-level First Occurrence (FO) records is also negative (Figure 2C; see Methods). The FO records are not necessarily sufficient evidence for oil escape, but rather are referenced for their potential utility of a paleo-geological metric for time (FO dates) in replacing the more abstract node number, which, if possible, would make oil composition a “global” molecular clock (discussed later). Our hope is that Figure 2C may inspire the further collection and utilization of more FO data to that aim.

### Oil escape adherence at the protein level

Here we show that “oil escape” occurs not only at the proteome level, but also at the individual protein composition level (which is evidenced by changes in oil content in groups of homologous, and later, orthologous, proteins over organism node space). Our “single protein” studies were performed on clusters of protein sequences homologous to “seed” protein domains listed in the SCOP database (v1.75, redundancy 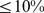) [21]. Within a cluster, each proteome was represented at most once, and homology was ascertained by BLAST-P's default values [22].

**Figure 3:**
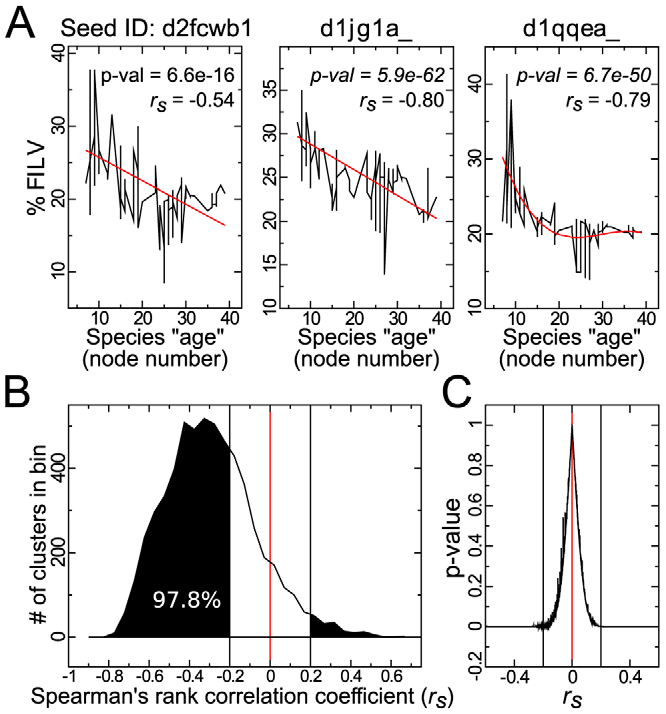
Most individual proteins display “oil escape”. Panel **A** shows examples of oil escape among three homologous clusters (seeded by SCOP protein domains listed in Section S2 in Text S1). Similarly, a large majority of the homologous clusters (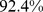 of the 5809 studied; see histogram **B**) undergo oil escape. Disregarding clusters with statistically irrelevant trends (defined by 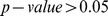 or 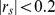; **C**), the percent of protein clusters displaying oil escape rises to 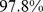 (we also obtained similar results for homologous protein clusters limited to each of the individual Ensemble databases [41] –bacteria, plants, fungi,metazoa; Figure S13 in Text S1). The clusters obtained were high in diversity, with an organism node number range of 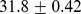 and average size of 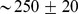 sequences, each sourced from distinct proteomes.

Interestingly, protein-resolution oil escapes, explicitly shown for three homologous clusters in Figure 3A and indicated by negative Spearman's correlation coefficients 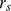, are not chance events as would be naïvely expected, but are the dominantly occurring trends among homologous clusters, with over 92.4% of the 5809 homologous protein clusters describing a decline in oil content over node space (Figure 3B). If we discount the statistically insignificant trends (i.e., if we omit those clusters showing two-tailed 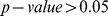 or 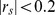, see Figure 3C), then the clusters displaying oil escape further increases to a compelling 97.8% of the 4518 viable clusters (also, analysis of clusters specific to individual Ensemble databases also resulted in qualitatively identical results; Figure S13 in Text S1). Figure 3B cannot be explained by the addition of more hydrophilic domains to a sequence over time, since 87.8% of the 4222 statistically significant (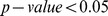) *size-homogenized* clusters display oil escape (here, size-homogeneity was ensured by culling all sequences that differ in length from the seed sequence by 20%; data not shown). Also, the decrease in oil content over node number is not expected to occur predominantly due to the addition of hydrophilic loops over node number as homologous clusters do not display a bias towards negative relationships between oil content and protein length *within* homologous clusters (Figure S9 in Text S1).

The homologous clusters of proteins used in Figure 3 contain protein pairs related by both orthologous and paralogous relationships (i.e., the proteins can either be related by direct descent, or by duplication and then descent, respectively), and the paralogous relatives in the cluster may cause inaccuracies in gene classification at the functional level [23], although our broad protein-fold resolution of inquiry may not elicit such issues. Still, the same study was carried out among smaller clusters of purely orthologous proteins (COGs database [24]), which also predominantly displayed oil escape among clusters describing significant trends (Figure S10B in Text S1); interestingly, including paralogs into the clusters (Figure S10A in Text S1) reduces the extent of oil escape observed, which indicates that Figure 3 may even be underestimating the extent of oil escape actually occurring.

These data, when consolidated, indicate that a large majority of proteins (homologs, orthologs, and individual domains) display marked oil escape, which reiterates the proteome-level studies in Figure 2, all of which indicate that more ancient proteomes (and so, by extrapolation, the LCA) display oilier residues (and, incidentally, fewer “specificity incurring” residues; see Figure S3 in Text S1) than the newcomers.

### Use of comparative methods

Despite the rough (coarse grained) nature of the utilized tree of life (ToL), the trend evident in Figure 2 is interesting, since a common trend–oil escape–appears to unite the behavior of all tested proteomes spanning the domains of life, which offers a unique glimpse into the LCA's molecular composition. This, however, requires that the character trait–oil content–be dependent on the species' ancestral history rather than on external factors (such as non-homogeneous changes in environmental temperatures), i.e., species oil content must display strong dependence on the topology of the tree of life, which is akin to phylogenetic dependence (PD) in purely phylogenetic trees [25], [26].

Using comparative methods, we confirm that while oil escape is dependent on the utilized ToL, that attribute is not sufficient to reiterate oil escape, which strengthens the notion of a directed Brownian evolution of oil content from an oily LCA. The dependence of a character state on a particular tree may be estimated using Pagel's 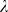 metric [25], [26], which normally ranges from 0 (no dependence) to 1 (strong dependence). We estimated (see method), from established methods [26], a strong bias of the pruned ToL on species oil content (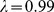; compared to 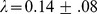 calculated from 1000 datasets with shuffled oil contents; Figure S14 in Text S1), which indicates that oil content is strongly dependent on a species' evolutionary history, and less likely swayed (or biased) by “other” (phylogenetically independent) forces not associated with shared history (e.g., non-homogeneous, evolutionary pressures).

It is also important to note that PD, while useful in precluding non-tree-based (or non systematic) biases in our trend, is not sufficient to reproduce the oil escape trend. This is easily evidenced by studying a “neutral drift” version of the original tree, where branches are modified to ensure that all species ages are identical (root to tip lengths are identical); while this tree does not display oil escape (given that all species ages are identical and not node number dependent), just like in the original ToL, high dependence is observed (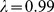; see Figure S14 in Text S1).

*Reconstruction of the LCA state*. From ancestral state estimation methods [27] (described in Methods), the “neutral drift” tree describes a random diffusion from an estimated ancestral state (LCA's estimated oil content 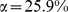, rate of change 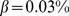 per node, and average error 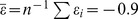; notation borrowed from [27]) that is very close to today's average proteome oil content (26.1%), while the original “speciation” tree describes a steady drift from an oily LCA (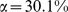; 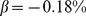 per node and 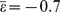), given the high 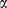 and substantially negative 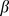. The “neutral tree” model of evolution by random diffusion is disregarded since the expected symmetric distribution of species about the predicted ancestral state is not observed (Figure S15 in Text S1). This leaves us with the “oil escape” model proposed in this paper (e.g., Figures 2,3), which may now be described as a “biased” Brownian motion in genome/proteome space at play particularly during speciation events.

## Discussion

### On tree completeness and bias

We discuss the two major problems that may potentially plague tree-related studies. First, the number of sequenced genomes (whose species populate the tree) is minuscule compared to the number of species observed in the biological universe (the incompleteness problem). Second, the genomes that are sequenced may be biased with respect to historic choice of model species, ease of handling new species, *et cetera* (the selection bias problem). Here we demonstrate how such biases do not disrupt our tree-related inferences.

**Figure 4:**
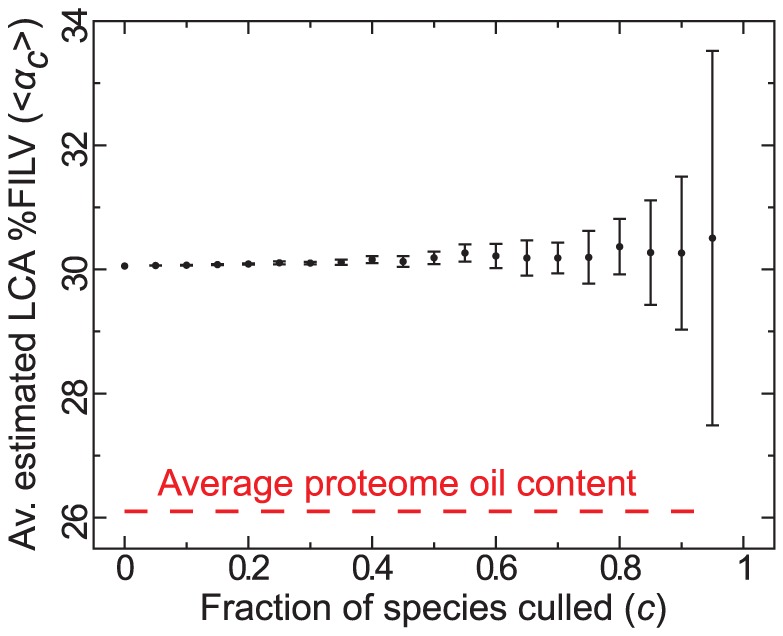
Estimated ancestral oil content vs. fraction (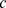) species culled. We obtained generalized least squares estimates of ancestral states using previously published methods [27], which indicate that the ancestral state estimates remain at the same average value (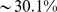) even when the species dataset is randomly culled down to 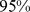 (100 randomly culled sets per 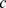). Interestingly, stastical relevance is only lost at 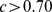, where the variance in estimated oil content (shown as error bars for 100 randomly culled sets per 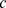) exceeds 0.25. This figure in expanded form is available in Figure S16 in Text S1.

*Incompleteness problem*. *(i)* It has been shown that the maximum likelihood comparative methodology used above is robust to incomplete phylogenies [26], which is reflected in our finding that the generalized least squares estimate for ancestral oil content is consistently reiterated (averaging at 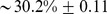) even when up to 70% of the species collection is randomly culled (Figure 4). *(ii)* the ToL utilized in our studies is obtained by pruning NCBI's ToL [28], [29] *without* collapsing single-children nodes [30], i.e., the node number (or shared path) of a species (or pair of species) within the pruned tree is equal to its corresponding value in the exhaustive NCBI tree (Section S6.1 in Text S1). Given the extensive species coverage in NCBI's taxonomy records (over 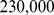 species are recorded [29]), the addition of newly sequenced species to our study will likely not modify the node number values for the extant species (or extant values 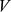), and so, adding new species proteomes to our relatively incomplete set of species will likely have no affect on the tree topology. These points indicate that while adding new proteomes to our analysis may improve the statistical relevance of our findings, data incompleteness is not expected to strongly disrupt our general inferences.

*Selection bias problem*. To ascertain the importance of selection bias (of sequenced species) in our results, we reduced the sampling bias by coarse graining our dataset to the genus level (averaging the node numbers and %FILV's within genera), which, while losing some statistical significance, reiterates the oil escape trend with negative %FILV vs. node number Spearman 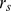 (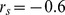; 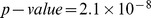; 76 data points). Our studies on randomly culled species sets (Figure 4, expanded in Figure S16 in Text S1) also indicate that bias due to over-sampling of related species is not likely to drastically modify the primary observation of oil escape, since randomized culling of species will likely reduce sampling bias (as oversampled relatives are more likely to be culled than unique species), which does not change our estimated ancestral oil content until very high culling frequencies are approached. Finally, it is important to note that the robustness of our data and analysis to incompleteness and selection bias may be due to the highly diverse dataset sourced from varied environments and node space (for example, the following node numbers host diverse species: node number 29 hosts the zebra fish, mosquito, and monkey; node number 23 hosts the louse and the platypus).

In conclusion to the previous sections, oil escape has been evidenced from simple regression analysis of whole proteomes (Figure 2), protein clusters (Figure 3), and by comparative methods. Finally, given that the biases due to incompleteness and sampling of the sequenced species and are not expected to negate/diminish the general trend of oil escape (see text, Figure 4, and Figures S15 and S16 in Text S1), we expect that oil escape is significant as a trend and merits discussion.

### Other trends

It must be noted that while other phylogenetically dependent traits *may* modulate oil escape, given the highly diverse nature of the tree, the possibility of explaining the entire trend with other niche based relationships/dependences is low. Figures S4 and S9 in Text S1 already indicate that oil escape can not be satisfactorily described by the increase of the protein length or the addition of loops or intrinsically disordered regions within globular proteins. Here, we will discuss trends in amino acid and composition previously reported in the literature to ensure that other potential relationships are also independent of the oil escape seen here, i.e., oil escape is a previously unreported trend.

**Figure 5:**
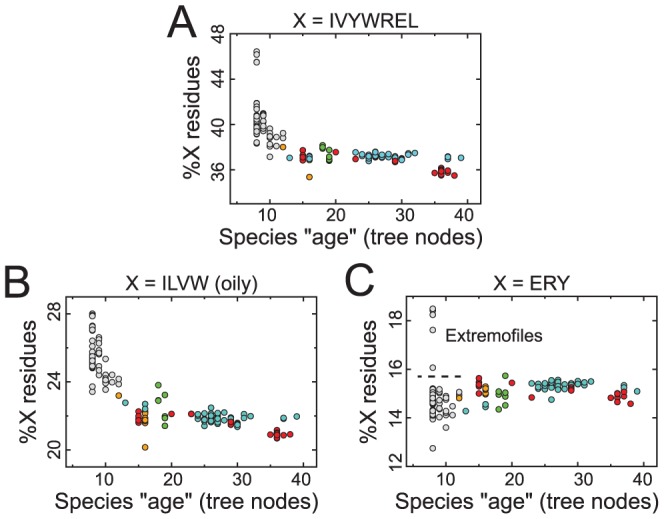
A universal reduction in %IVYWREL over time is a result of strongly reducing oil content. At first glance, it appears as though the combined fraction of IVYWREL amino acids in a proteome (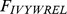), which is a correlate of prokaryote Optimal Growth Temperature in prokaryotes [31], is also inversely correlated with node number or evolutionary time for both prokaryotes and eukaryotes (**A**; 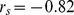, 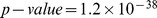, 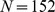). However, this effect is primarily caused by the hydrophobic residues of the set (IVWL; **B**; 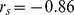,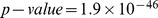), while the rest of the amino acids (ERY) show the opposite mildly *increasing* relationship with respect to node number (**C**; 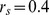,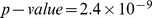). We can more certainly conclude that the trend in 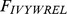 seen in (**A**) is only a result of “oil escape” given the following information: (i) both groups (IVWL and ERY) are required to be positively correlated in order for IVYWREL to be predictive of OGT (Figure S5A–D in Text S1), which is not the case (for each proteome, the Spearman correlation between 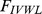 and 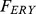 is 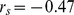, 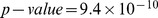; also see **B** and **C**), and (ii) oil content alone is not well correlated with OGT (Figure S5E,F in Text S1), and so the two trends of “oil escape” and decrease in OGT can easily be unambiguously distinguished. The extremofile outliers in (**C**) are: *Pyrococcus* (*P*) *abyssi* (abscissa value: 8, ordinate value: 18.4), *P kodakaraensis* (8, 18.5), *P furiosus* (8, 18.2), *P horikoshii* (8, 17.6), and *Bacillus halodurans* (8, 16.1).

*(i) Optimal growth temperatures (OGT)*. A previous report has established a strong positive correlation (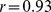) between proteome %IVYWREL (single-letter amino acid codes used) and a prokaryote's optimal growth temperature or OGT [31] (the relationship is reiterated in Figure S5A in Text S1). At first glance, species age (node number) and proteome %IVYWREL appears to also correlate well (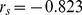, 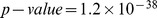; see Figure 5A), which could have indicated an interesting relationship between change in living conditions (temperature) and the progression of complex lifeforms. However, the utility of %IVYWREL as an OGT “thermometer” is contingent upon equal representation (and positive correlation) of both high and low hydrophobic amino acid groups within IVYWREL (i.e., %ILVW and %ERY are also expected to positively correlate with each other; see Figure S5B–F in Text S1); given the lack of positive correlation between %ILVW and %ERY (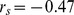, 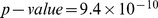), the trend in Figure 5A is merely a result of oil escape, which is also indicated by the opposing relationships in Figure 5B,C; the trend in Figure 5A appears to be caused primarily by oil escape.

*(ii) Oil escape is not a spurious relationship*. With the advent of genome and proteome databases, a number of additional interrelationships associated with genome/proteome composition have been reported. Of particular interest to the focus of this paper (oil escape) are the reported relationships between %GC content and genome size [32], organismal complexity and genome/proteome length [33], and %GC and hydrophobicity [34]–[38]. It is important to exclude the possibility that oil escape may be a consequence of potentially more strongly evidenced relationships. For example, could %GC content, which is known to be a correlate with hydrophobicity [39], [40], be a stronger correlate with node number? Or are other sets of correlations transitively inducing the effect of oil escape?

**Figure 6:**
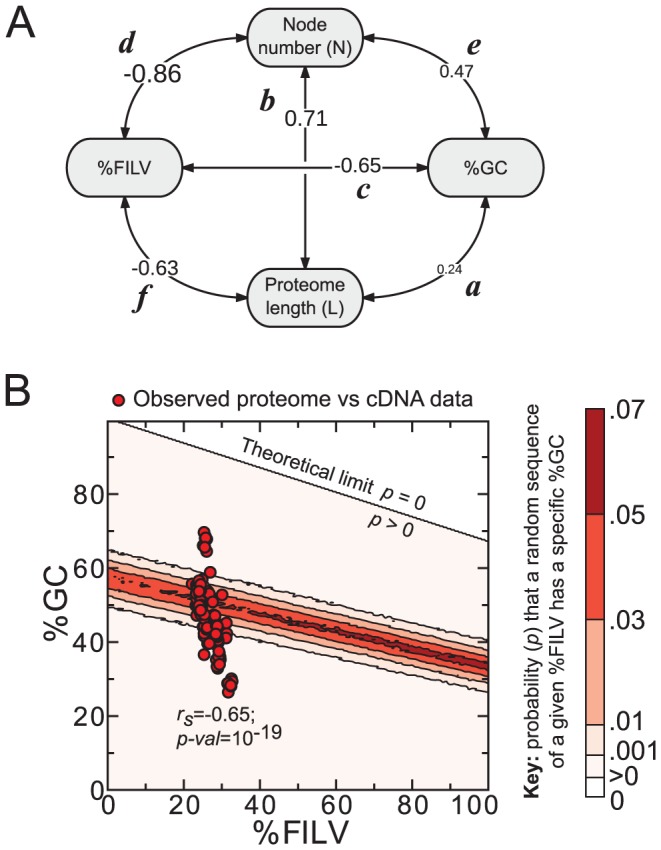
(A) Oil escape is independent of other relationships. All-versus-all Spearman correlation coefficients were calculated for all possible pairs involving *(i)* species cDNA %GC, *(ii)* proteome %FILV, *(iii)* node number 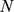 and *(iv)* proteome length 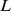 (individual graphs shown in Figure S12B in Text S1). The results indicate that oil escape (***d***) can not be caused by the other variables (see discussion). Relationships between other variables as well as effects of population size on compositions are also discussed in the text. (**B**) **Change in %GC per node number may be an independent trend.** Finally, despite the strong correlation between %FILV and %GC (***d***), the relatively strong relationship between %GC and node number is expected to be independent of oil escape due to the incongruence between expected (contour plot in **B**) and observed correlations (observation shown as red circles in **B**). P-values for each of the regressions ***(a)*** through ***(d)*** are all statistically acceptable with values approximating 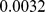, 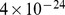, 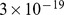, 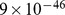, 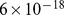, and 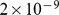, respectively.

In order to tackle these questions, we calculated all possible relationships between species cDNA %GC (also obtained from Ensembl genomes [41]), proteome oil content (%FILV), species node number (N), and proteome length L (a proxy for organismal complexity in prokaryotes [33], [42]–[44]), which can be described as a complete correlation network (Figure 6A) where labeled edges (***a***–***f***) indicate Spearman correlation coefficients between properties shown as nodes. It is evident from this graph that oil escape (***d***) displays a Spearman correlation that is highest in magnitude (and consequently, also lowest in p-value). While this may hint at the independence of oil escape from other variables, only statistical tests [45] are able to to strike out the possibility that other variables in Figure 6A are incapable of causing oil escape. This requires the introduction of a statistical criterion that establishes transitivity (and hence the possibility of causality [46]) to a triplet of variables [45], [47]. Given three variables 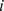, 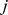 and 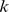, and their Spearman or Pearson correlation coefficients 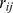, 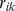, and 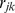, one can show that if 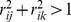, then 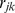
's sign must be commensurate to the two other relationships, i.e., 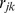
's sign must equal that of 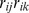 [45], [47]. However, when this criterion is not met, the trend between 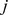 and 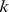 (indicated by 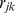) can not be caused by the two other correlations. Therefore, while causality may be incapable of being proven using correlations, the *lack* of causality or transitivity can be shown in some situations. Given this criterion, we can conclude that none of the observed adjacent relationships in Figure 6A are able to *cause* oil escape ***d*** (since, from Figure 6A, both 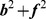 and 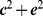 are less than 1).

*(iii) GC content vs node number: a possibly independent trend*. So far, we have shown that oil escape (***d***) is likely not caused by any other pair of correlations in Figure 6A. However, we are still left with the question of which of the remaining correlations are caused by others. For example, while a strong relationship is indicated between species %FILV and %GC (***c***), the relationship is possibly not direct, which we can be shown by a practical implementation of “proof by contradiction”. First, we assume that a non-spurious negative relationship exists between oil content and %GC, i.e., the expected biases [39], [40] in the codon table (or genetic structure) [48], [49] are the main causes of the %FILV-%GC relationship. With this assumption, we are able to quantify the expected %FILV-%GC relationship by generating, for each 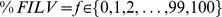, 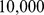 randomly assembled genes that each code for a hundred amino acid peptide with oil content 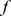. From these pairs of gene %GC and corresponding protein %FILV, we created a probability distribution that is represented as a contour plot in Figure 6B (the additional “limiting” contour line dividing the impossible, 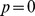, from possible, 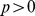, was added by analyzing the extremum possibilities from the codon table). Importantly, the relationship observed between proteome %FILV and cDNA %GC (red circles in Figure 6B) displays no correspondence with the expected probability distribution. Also, when sampling generated sequences taken between the observed 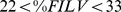 range, the expected Spearman correlation between %FILV and %GC is 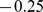 (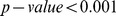), which indicates a relatively weak relationship (as indicated by the broad contour distribution in Figure 6B) that is significantly distinct from the observed 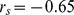 (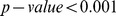). These incongruencies between expected and observed relationships indicate that the observed relationship between %FILV (or hydrophobicity [35]) and %GC is not directly caused by the codon table, and, therefore, the relationship between %GC and node number observed is independent of oil escape. We leave the exploration of the cause of trend (***e***) to future research.

*(iv) A note on population size*. Effective population size is an important factor in population genetics [50], particularly because deleterious mutations that result in offspring of lower fitness are more likely to be maintained in smaller effective population sizes [51]. It is therefore important to establish whether population size is capable of affecting changes in proteome and genome composition. Given the sparse statistics on effective population sizes, we chose to use proteome length (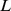) as an inverse metric for population size, which is strongly indicated in previous reports [33], [42]–[44]. It is important to recognize that 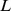 can be utilized to relate population size to %FILV or %GC only if the relationships display transitivity [45], [47] via 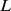, which is a property that still requires verification in future studies (in the absence of transitivity, all relationships involving *population size* must be discounted). The first correlation that we can verify is the expected positive relationship [33], [42], [44] between organismal complexity (node number 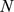) and proteome length 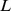 (***b***), and, assuming transitivity, a negative relationship between complexity (via 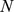) and population size (via 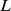) [33], [42]–[44].

We now direct our attention to the relationship between population size and %GC. Assuming transitivity, %GC content displays only a weak correlation with population size through 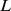 (Figure 6A ***a***). While the actual trend (Figure S12B in Text S1) does not qualitatively reproduce the non-monotonic relationship between between %GC and population size reported from simulations [51], the trend (especially when calculated exclusively for bacteria studies, where 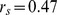 (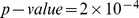) do match the %GC versus 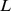 studies in bacteria [32]. The reason for the incongruence with simulation [51] may be due to the possible lack of transitivity of population size and %GC through 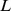. However, assuming transitivity, our data supports the notion that genomes with high AT content display a fitness advantage compared to genomes with low AT content; this implies a mutational bias towards high AT content, which is universally observed even in high-GC bacteria [52].

Finally, we will evidence that, despite the apparent correlations, %FILV is not likely to be strongly or directly dependent on 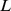 and therefore (assuming transitivity) population size. While %FILV does display a negative *correlation* with 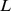, there is high probability that the result is spurious and caused by relationships ***b*** and ***d***, since 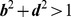, making ***b*** and ***d*** transitive [45], [47], while 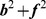 and 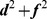 are both less than 1. The potentially causal relationship is also indicated by the stronger correlation of 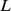 with 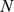 (Figure 6A ***b***) and a high phylogenetic dependence of 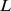 on the tree of life and therefore node number (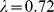, where 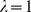 indicates the strongest dependence and trees with shuffled leaf attributes yield 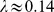). Therefore, our studies are unable to discern a strong relationship between species population size and proteome %FILV. The lack of this relationship is possibly due to the lack of transitivity between the involved proxy variables. Regardless, the measure of fitness of a protein is not likely to be directly related to the thermodynamic stability of the protein, but to the capacity to maintain a particular near-ground-state ensemble of structures, which is not strongly dependent on only hydrophobicity, but dependent on both hydrophobicity and the buried polar interactions that impart greater folding specificity [53]–[55], and defray, in part, the entropic cost of hydrophobic collapse [56].

To conclude this section, we find that oil escape (%FILV vs node number) is indeed a significant relationship that is not expected to be caused by other variables such as %GC content, genome length (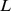), population size (through 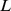), and optimal growth temperatures.

### Oil escape appears to be asymptotically slowing down

Besides displaying a strong monotonic trend (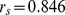), the scatter plot that describes oil escape (Figure 2B) is also modeled well (with correlation coefficient 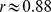 for two instances described below) by the following asymptotic closed form:
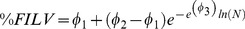
(1)Here, 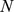, 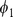, 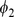, and 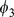 are the node number, asymptote (in 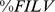), ordinate-intersect, and “rate constant” of the trend, respectively. Keeping all 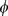
's variable, and fitting to the data in Figure 2B, we obtained the asymptote 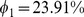 (with 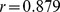; Section S4 in Text S1), which, despite the coarse grained quality of node number (and the tree of life), is remarkably close to the expected % of codons (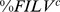) that code for oily residues (from the universal codon table, 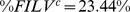). Also, constraining 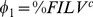 still results in a similarly high 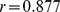 (Section S4 in Text S1). These findings strengthen the idea of asymptotic decline in oil content, where the LCA's proteome originated as more oily than expected from sequence entropy considerations (if one expects equal distributions of codon usage), following which later organisms asymptotically approach more moderate values (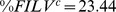). It is important to note that, for our data, alternative genetic codon tables do not change the value of 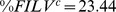 [48], [49], making this a universal maximum for sequence entropy. As a footnote, while one of the six alternative nuclear codon tables has a lower 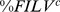 of 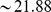, this “outlier table” is only utilized by a subset of the *Candida* genus of yeasts that is not utilized/represented in our data. Finally, the nearing of later proteomes' oil content towards the “maximal entropy” value (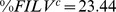) may have interesting implications regarding changes in the rates of molecular evolution.

### Oil escape is a passive (entropically driven) drift

The list of species used in our proteome/protein-level studies are diverse, sourced from all domains of life and displays a large range of cellular makeup, body types, biochemistries, evolutionary niches, etc. Given this diversity, we conjecture that oil escape is not driven by an adaptive pressure, as the niche diversity precludes such a broad spectrum pressure. Additionally, given that previous trends such as dependence of proteome composition on oil content do not explain the oil escape trend, we provide a hypothesis based not on extant adaptive pressures but on the features of the last common ancestor (LCA). It is important to note that oil escape must be a trace (fossil) drift from the LCA which would have predominantly begun *after* the production of the first fit proteome with acceptable but relatively high oil content (this is especially the case as all observed oil contents today are expected to be within the range of “acceptable” oil content, which is relatively quite broad, 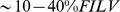; Figure S4 in Text S1). The subsequent reduction in oil content should be considered to be more of a passive drift driven by the impetus to maximize sequence information entropy through evolutionary time, i.e., adaptive pressures and neutral drift would not be driving forces in oil escape, although coupling of the passive drift with either phenomenon is possible. Still, oil escape is not likely to be coupled to neutral drift, which can primarily account for substitutions of nearly-neutral fitness. Alternatively, oil escape may easily be coupled to mutations occurring during speciation events, partly due to the putative increase in substitution rates [13]–[15], [57]–[64], fixation probabilities [65], and hitchhiking [66]–[68] of composition-changing mutations with adaptive mutations occurring during speciation events. It is important to reiterate that while oil escape itself might be coupled to adaptive processes, the impetus to shift the oil content of a sequence is driven by the need to maximize information entropy and not adaptive forces.

### Oil escape as a molecular clock

This passive drift is slow in effect, as change in oil content (and most other compositions, like charge content) is impeded by the biochemical requirements of a protein (Sections S1.2 and S1.4 in Text S1) and the proteome's massive size (Section S1.1 in Text S1), and therefore is not expected to be “washed away” by a random mutational walk over billions of years (Section S1.1 in Text S1). This ensures the persistence of oil escape even billions of years after the initiation of the trend, i.e., one may consider oil escape as a trace fossil.

These considerations indicate that the oil escape trend summarized in Figure 2B appears to be a rare, universal “reaction coordinate” for evolution, adhered to (at least roughly) by all species tested ranging from bacteria to animals, which may serve as a unique *composition*-level or “global” molecular clock that, if calibrated, may augment the utility of sequence-based “local” molecular clocks [69]. The calibration itself would require more species-level paleobiological first occurrence data for species whose genomes are sequenced. Figure 2C, while rough and statistically sub-optimal, provides a proof of concept for such a calibration project.

It is also important to note that features such as variable substitution rates [70], [71] may pose inherent problems to the utility of oil escape as a molecular clock. However, the finding of a genome “core” that describes, even for bacteria, genes with conservative substitution rates, lower involvement in HGTs, and higher reliability in reconstructing robust trees [72]–[80] indicates that the selection of appropriate protein collections for utility in the global molecular clock may ameliorate a number of inherent problems associated with a global molecular clock. Finally, oil escape is a function of the number of substitutions associated with node number and therefore speciation, making it distinct from neutral molecular evolution. Whether mutations associated with speciation events can be used as a metric for evolutionary age remains to be seen and warrants further research.

### Effects of horizontal gene transfer and recombination

The possibility of recombination and horizontal gene transfer (HGT) [81]–[84] between species indicates that distinct genes may have distinct evolutionary histories, resulting in the tree of life being rendered, in one extreme formulation [72], [73], as a *network* of life [74]. Such a free exchange of genes between species would mean a much quicker equilibration of any compositional inequities between species and hence a drastic degradation of the oil escape signal. However, the following points indicate that HGTs are not significantly detrimental to the oil escape signal: (a) complex organisms, while not completely immune to HGT, are generally not affected by this mode of evolution [85], (b) a large percent (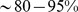) of the genes/proteins that are crucial to the core functioning of an organism are not replaced or affected by HGT [72]–[74], while “accessory” or niche-associated genes partake most in HGTs [74]. The observation of oil escape in both bacterial and complex organism databases (hosting fungi, plants, and metazoa) and orthologous groups spanning all domains of life (mimicking core genes/proteins) indicates that, while horizontal gene transfer may contribute to the noise in the oil escape signal, the complete degradation of the signal is not evident.

While tree reconstructions based on one or few genes may be prone to errors due to recombination and HGT events, utilization of a high volume of independent reference points (such as a whole proteins) results in trees that are robust to recombination and HGT events [78]–[80]. This indicates that the NCBI tree of life, which is expertly compiled from a highly diverse set of phylogenetic and taxonomic data, has a very low chance of being drastically affected by HGT and recombination.

### Rise of disordered proteins

Intrinsically disordered proteins (IDPs) are low-hydrophobicity proteins that remain unfolded for most of their existence [86]. Recently, a strong connection has been recognized between the rise of complex organisms and the increase in the incidence of IDPs in genomes [87]. The driving forces behind both the increase in organismal complexity and the increase in the incidence of IDPs are still unknown, although they are believed to not be driven by adaptive evolution [88]. It is interesting then that oil escape, when considered as a shift in Gaussian *distributions* of oil content within a proteome, is able to predict the gradual increase in IDP incidence by the gradual decrease in oil content. Particularly, the shift of the distribution of oil content from prokaryote to eukaryote (as observed in Figure S11 in Text S1) appears to push the left tail/fringe of the distribution beyond a hydrophilicity threshold that allows for IDPs to exist [86]. Also, given that bacterial proteomes alone also exhibit statistically-significant oil escape (Figure S1 in Text S1), we can conclude that oil escape is not *caused* by the increase in IDPs (and hence organismic complexity), while the increase in the incidence of IDPs may be driven by oil escape. To our knowledge, oil escape from a relatively oily ancestor is the first non-adaptive explanation of the emergence of IDPs, which fits well with the assertion that no adaptive processes can account for the increase in organismal complexity [88].

### Is molecular evolution irreversible?

Given the enormity of sequence space, the chances of reverting to a distant common ancestor are abysmal, a notion that helps paint a one-directional picture of molecular and hence organismal evolution (see, e.g., work by Bridgham *et al.* [89]). To add to this unidirectionality, Jordan *et al.* have provided an interesting observation [5] indicating that there might even be a directionality or “irreversibility” to the *types* of mutations incurred within a protein (e.g., they reported that 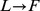 residue substitutions occur more often than 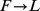 substitutions), which contradicts the general notion of reversibility or symmetry in point mutations (where 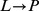 and 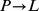 substitutions are equally probable and the substitution matrix is symmetric). While this paper has been challenged [7]–[10] primarily due to the reconstruction methods utilized within the paper, we find from a much simpler methodology that such a directionality *may* exist, albeit in not exactly the same form as reported previously (see Figure S7 in Text S1).

### Our last common ancestor may not be the most “likely” one

Finally, to underline the dangers involved in extrapolating from present day sequences to a putative common ancestor by statistical methods, it is important to recognize that the LCA's proteome composition may have not been the most “likely” sequence from a sequence entropy standpoint; as indicated in Figure 2B, the ancestral sequence may have high oil content, indicating a less-likely *low* sequence entropy. This raises an interesting and unforeseen pitfall in the backwards extrapolation of the last common ancestor's sequence using methods involving maximum likelihood methods: while backwards-extrapolation may hold ground when used in less diverged groups, when extending further back in evolutionary time, we might be presently exploring a biophysically meaningful but historically meaningless section of sequence space. Such a pitfall may be circumvented by applying a “field” (or mutational bias) to a prediction method.

### Concluding remarks

Looking forward, the notion of oil escape has varied implications. Most importantly, oil escape–a universal trend relating oil content to tree node number–evidences the emergence of all known organisms from an oily last common ancestor, and provides a biophysical explanation to the emergence of intrinsically disordered proteins [86]. Additionally, the existence of at least two seemingly *independent* constraints on genome/proteome composition (evidenced in Figure 6) indicates a robustness of evolving genomes placed under multiple *universal* constraints. Finally, the universal fossil trend in proteomes today, aside from serving as the first potential *global* molecular clock, provides glimpses into the earliest proteome, and may provide validity to the possible irreversibility of evolution [5].

## Methods

### Databases used

The predicted proteome and cDNA sequences used were both obtained from Ensemble genome databases [41] which, at the time of procurement, hosted 272 diverse proteomes belonging to 152 distinct species sourced from all domains of life (listed in Section S5 in Text S1). Both proteome and cDNA sequences were obtained from all predicted genes, known or otherwise [41]. Only amino acids that belong to the natural 20 amino acid repertoire were used in our proteome calculations. Similarly, only nucleotides A, T, G and C were used in our cDNA calculations.

### A metric for species evolvedness

Expanding on the general idea that species that are less diverged from the last common ancestor (e.g., bacteria) possess older proteins/proteomes than more diverged species (e.g., fruit fly) [13]–[15], [20], we define a species/proteome's modernity or newness as the minimum number of nodes– *node number*–separating the species or organism from the LCA (root) in the tree of life (ToL). The pruned tree of life (Section S6.2 in Text S1), containing all studied species (Section S5 in Text S1), was obtained using NCBI Taxonomy's Common Tree algorithm [28], [29] (accessed from the interactive tree of life [30] with the selected option of leaving “internal nodes expanded”). This tree classification system is based on expert but heuristic gathering of phylogenetic, taxonomic and other biological information (excerpt from the NCBI taxonomy website: “…[The] database does not follow a single taxonomic treatise but rather attempts to incorporate phylogenetic and taxonomic knowledge from a variety of sources, including the published literature, web-based databases, and the advice of sequence submitters and outside taxonomy experts”; http://www.ncbi.nlm.nih.gov/Taxonomy/taxonomyhome.html/index.cgi?chapter=howcite), and is therefore not biased towards specific classification methods. For example, while distinct issues are associated with ignoring horizontal gene transfer [74] and recombination [81] in phylogenetic tree reconstruction, a robust and faithful tracing of lineages is possible with the utilization of larger sequence datasets sourced from whole proteomes [78]–[80].

### First occurrence (FO) records

The paleobiological FO records (listed in parenthesis in Section S5 in Text S1) are obtained from the online paleobiology database (pbdb.org). Due to the relative sparseness of the online records, we associated a species in our collection to its *genus* FO record, which makes this metric only a coarse grained indicator of evolutionary age (e.g., the mosquito species *Anopheles gambiae*, was linked to the genus *Anopheles*, which has the FO date of 23.030 Ma or million years before current date).

### Estimating the 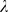 metric phylogenetic dependence

This section summarizes the phylogenetic method introduced by Pagel [25] and expanded by Freckleton *et al.* [26]. The ToL may be described as a variance-covariance matrix 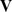, where elements 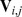 denote the evolutionary path lengths shared between species 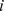 and 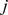 (so, 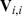 indicates species 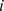
's node number in the original tree and a constant in the “neutral drift” tree). Also, we may set a (column) vector 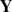 which contains the character traits of the species in the tree (in our case, 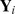 is equal to the oil content of species 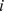). The extent to which a given character state 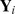 depends on its position in the phylogeny may be assessed using an off diagonal multiplier 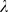, where the variance-covariance matrix is transformed to 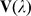 by multiplying all off diagonal elements (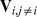) by 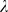 where normally 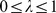. The estimated 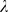 will be that which maximizes the likelihood function 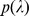 (obtained from Equation 4 in Freckleton et al. [26]) for a given 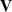 (tree) and 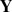 (character set; for examples of such 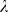-searches, see Figure S14 in Text S1). A maximum likelihood estimate of 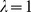 would indicate that the character state is evolving according to the Brownian model of evolution on the phylogeny, while 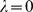 (where only the diagonal elements in 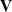 remain non-zero) indicates a character trait that is independent of the given phylogeny and shared histories.

For convenience, Equation 4 in Freckleton et al., which is a joint-normal probability density, is repeated here:
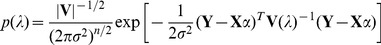
(2)where 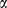 is the character state at operational time 0, 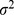 is the variance of the Brownian noise introduced in the character state per time unit, and 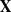 is an 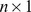 “design matrix” of ones which describes a Brownian mode of evolution (setting 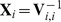 or 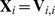, which describes a simplified “biased” Brownian model of evolution, does not significantly change our estimated 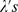). The maximum likelihood estimate for 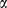 is
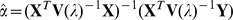
and the unbiased (restricted maximum likelihood) estimate for variance 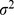 is
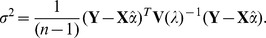
By maximizing Equation 2, our maximum likelihood 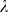 for a given tree may be estimated. For both the original and “nearly neutral” trees we obtained 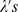 of 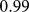 (Figure S14 in Text S1), indicating strong phylogenetic dependence. Note that the estimated 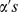 are likely not accurate, since it's value is directly controlled by the “design matrix” 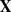, whose model (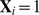) is over-simplified to an unbiased Brownian diffusion about the ancestral state 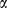.

### Estimating the ancestral oil content

We use a previously described generalized mean squares model of evolution [27], which describes the character state 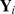 of species 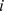 by 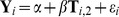, where 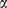 is the character state of the ancestor (LCA) at operational time 0, 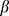 is the estimated rate of change of the character state per operational time unit (e.g., node number), 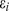 is the random error, and 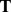 is an 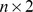 matrix whose first column elements all equal 1 and the second column elements depicts the species operational time/node number (i.e., 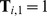 and 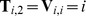
's operational time or node number) [27]. From the generalized least squares method [27], we can estimate both 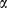 and 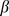 by solving for
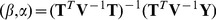
(3)where 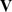 is the variance-covariance matrix of the given tree (as above; i.e., 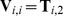). Also, the error 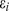 for species 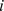 may then be obtained from 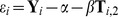.

While the validity of the previous 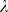-method of obtaining 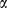 was predicated by the choice of the design matrix *and* the statistical inaccuracies of the tree (caused by it's coarse-grained nature), the current reconstruction method does away with the Brownian diffusion model, and so is only dependent on statistical inaccuracies of the tree. However, given that, it is safer to use such 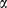 estimates as qualitative checks for hypothesis/data validity (e.g., in Figure 4) rather than an absolute prediction of the ancestral state.

## Supporting Information

Text S1 **Supporting discussions, figures and data.** This document contains discussions and additional analysis that support the findings and claims made in the main article. Additionally, the complete list of species utilized along with the tree of life utilized are listed at the end.
(PDF)
