# Supplementary material for: A Universal Trend among Proteomes Indicates an Oily Last Common Ancestor
Source: PLoS Comput Biol. 2012 Dec 27;8(12):e1002839. doi: 10.1371/journal.pcbi.1002839 (PMC3531291; doi:10.1371/journal.pcbi.1002839)
Supplement: Text S1 — Supporting discussions, figures and data. This document contains discussions and additional analysis that support the findings and claims made in the main article. Additionally, the complete list of species utilized along with the tree of life utilized are listed at the end. (PDF) [file pcbi.1002839.s001.pdf]

Text S1. Supporting information to: "A universal trend among proteomes indicates an oily last common ancestor"

Ranjan V. Mannige[1,2,3,4*], Charles L. Brooks III[2] and Eugene I. Shakhnovich[1]
[1]Department of Chemistry and Chemical Biology, Harvard University, Cambridge, MA, USA
[2]Departments of Chemistry and Biophysics,University of Michigan, Ann Arbor, MI, USA
[3]Department of Molecular Biology, The Scripps Research Institute, La Jolla, CA, USA
[4]Center for Theoretical Biological Physics, UC San Diego, La Jolla, CA, USA
[*]To whom correspondence should be addressed; E-mail:
rvmannige@lbl.gov

S1 Discussions regarding molecular evolution and proteome composition change.

The following three subsections will discuss the following three points respectively: (i) even through every organism today is expected to have existed in *some* form since the emergence of life (Darwin's common descent axiom), not all proteomes have evolved/mutated to the the same extent since the last common ancestor (LCA); speciation events account for a large number of sequence substitutions; (ii) probability of oil-content-changing substitutions are expected to occur more often during speciation events; (iii) in absence of an entropic drive to change oil content, one would need an unacceptably large number of random mutations to observe the range of oil contents observed in our dataset.

S1.1 A sequence's oil content is expected to change at a glacial pace compared to the molecular evolution that drives it; a continual random walk in sequence space for billions of years can not explain the range of protein hydrophobicity observed

As an alternative (null) hypothesis to the oil escape described in the paper, let us assume that the (8%) range in oil content observed in the paper (Fig. 2) is due to a random drift in sequence space since the LCA. We can show, by modeling the evolution of the proteome by a random walk, that this null hypothesis is very unlikely to have occurred.

Take a proteome of size N, with H number of hydrophobic residues ($0 \leq H \leq N$). Also, assume that any mutation independently changes the hydrophobicity (H) by ± 1 amino acid. Each successive accrued mutation may then be considered as a random walk on an integer number line (shown below) where the current position is the hydrophobicity of the proteome.

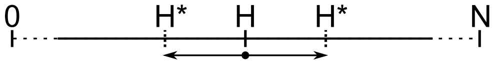

H = number of hydrophobic amino acids in proteome.

Assuming that M point mutations have been accrued, the change in the number of hydrophobic residues in the proteome, ΔH, will be approximately of the order of the $\sqrt{M}$ (obtained from theory on random walks on integer number lines), i.e.,

$\Delta H \approx \sqrt{M}$

So, the change in fraction (or percent) hydrophobic residue in the proteome will be relatable to the number of accumulated mutations per amino acid position:

$$\frac{\Delta H\%}{100} \; or \; \Delta H_{fraction} = \frac{\Delta H}{N} \approx \frac{\sqrt{M}}{N}$$

Now, assuming a proteome size of $N = 2,000,000$ (which is the size scale of a proteome belonging to a large bacterium such as *Mycobacterium smegmatis*), given the equation above, we would require about $(N \times 0.08)^2 = 25.8 \times 10^9$ number of random mutations per proteome (or $12,800$ mutations per amino acid position) to shift the hydrophobicity of the proteome by the observed 8%. Given the acceptable average mutational rate of 10^{-9} mutations per amino acid position per year or 1 mutation per position per one billion years (a commonly reported magnitude[1]), we would expect a random walk to produce an 8% drift in hydrophobicity (for $N = 2,000,000$) only after roughly 12.8×10^{12} or 12.8 trillion years of evolution, which is substantially larger than the expected age of the universe, much less the expected age of earth.

Although the example above uses a specific value for proteome size (N), plugging in other values for N in the above equation will provide similar results, indicating the implausibility of attaining the range of hydrophobicity by simple random mutational diffusion. For example, a high average mutation rate of 6×10^{-9} brings the number of years required to obtain an 8% shift in hydrophobicity to a still high 2.1 trillion years. For the present N, one would need a substitution rate of 4.26×10^{-6} to cause an 8% shift in hydrophobicity, which is far from the observed regime of $10^{-}9$ amino acid substitutions per site per year; the observed change in % hydrophobicity ($\Delta H\%$) can not be attributed to or caused by random mutational drift in the sequence, which raises the plausibility of drift by oil escape. The following subsection further strengthens this claim.

More/empirical problems with randomly changing a proteome's H. There are also other considerations that further diminish the effect of random point mutations on drift in hydrophobic content:

(A) the random walk in H (described above) required that mutations are always "non-synonymous" (i.e., they *must* change H). However, this move set does not consider "synonymous" mutations, where one hydrophobic residue is replaced with another (and the same for non-hydrophobic residues), which will drastically increase the number of mutations required to obtain a translation of 1 unit in the number line above (since many "walks" will be ineffective in translating H). For example, if we consider only F,I,L and V as hydrophobic amino acids (chosen since %FILV was our metric for hydrophobicity in the manuscript), then an attempted mutation will be a hydrophobicity modifying mutation only 32% of the time.

(B) As evident in the sequence substitution matrices (Table S1), mutations that maintain proteome (oil or charge) composition are much likely to occur (and be kept for posterity) than mutations that modify oil/charge content.

Both (A) and (B) indicate that the actual extent to which random accumulated mutations or sequence "drift" affects oil content will be even lower than those estimated by a simple random walk in H space (since more mutations are required in order to shift a proteome's H by even 1 unit on the number scale above).

In conclusion, the composition shifting substitutions within a genome are likely not made during neutral drift, i.e., g's contribution to the composition change by substitution that we term as "oil escape" is expected to be minimal compared to the substitutions inncurred during the speciation events due to increased substitution rates,[2–12] hitchhiking,[13–15] and presumably reduced population sizes (and high fixation probabilities)[16] during speciation events.

S1.2 Mutations tend to favor the preservation of oil and charge content in a sequence.

	Average log odds for a type of mutation:				
	Random mutation	Composition shifting mutation		Composition preserving mutation	
Substitution matrix used	**X** $\leftrightarrow$ **X**	**H**$'$ $\leftrightarrow$ **H**	**C**$'$ $\leftrightarrow$ **C**	**H** $\Leftrightarrow$ **H**	**C** $\Leftrightarrow$ **C**
Blosum30[17]	-0.0215	-0.3132	-0.1816	0.7000	0.5800
Blosum40	-0.0523	-0.5263	-0.3059	0.9250	0.8750
Blosum50	-0.1191	-0.8158	-0.5088	1.0000	1.0000
Blosum62	-0.2605	-1.0197	-0.6579	1.1500	1.1500
Blosum70	-0.3157	-1.2039	-0.8289	1.1500	1.1500
Blosum80	-0.2481	-1.3246	-0.9211	1.1333	1.1667
Blosum85	-0.4077	-1.4408	-1.0329	1.1500	1.2000
Blosum90	-0.4444	-1.5197	-1.1118	1.1500	1.1500

Table S1: **Historically, fixed mutations that preserve oil/charge content are much more probable than those that do not.** Proteome composition-maintaining mutations are highly probable, which is indicated by a positive average log-odds score (see equation below; each log odds value was obtained from matrices found on the NCBI FTP database at ftp://ftp.ncbi.nih.gov/blast/matrices/). Conversely, proteome composition-shifting mutations appear to be even more improbable than mutations that appear randomly. This indicates that, a random drift of sequence space is under active pressure to maintain proteome composition (almost certainly due to the biophysical requirement of maintaining protein structure/function). Note that while the Blosum matrices are general indicators of trends of acceptable substitutions, such a matrix can not represent all sequence evolution situations given the heterogeniety in rates of nucleotide substitution.[18,19]

AMINO ACID KEYS: **X** is the set of all possible amino acids, the hydrophobic amino acids **H** $\in [F, I, L, V]$ (the top four most hydrophobic residues as per the Kyte-Doolittle scale), and the charged amino acids **C** $\in [E, R, D, K]$, while **H**$'$ and **C**$'$ are all amino acids not including **H** and **C**, respectively.

EQUATION: The log-odds of substitution (M_{ij} in bit units), for any two amino acids i, j was obtained from published Blosum matrices[17] and normalized to represent bit units; so, $M_{ij} = log_2\left(P_{ij}/q_i q_j\right)$, where q_i is the natural frequency of amino acid i in sequences, P_{ij} is the probability of the two amino acids i and j replacing each other at a homologous position. The odds of a mutation may be obtained from the expression $2^{M_{ij}}$.

The various Blosum[17] substitution matrices (obtained from the NCBI FTP database ftp://ftp.ncbi.nih.gov/blast/matrices/) indicate that mutations that preserve the proteome/sequence's % hydrophobic content (**H**) (and % charge content, **C**) are much more probable than those that cause fluctuations in oil or charge content. Notably, **H** and **C** modifying mutations are not even marginally tolerated (as indicated by their lower average log odds score than even the expected log odds score for a randomly picked amino acid pair).

S1.3 Rates of molecular evolution (by nucleotide substitutions) and speciation events are linked (i.e., node number $\propto$ number of accumulated substitutions).

Summary:

- It has been shown that speciation events are concomitant with an increased rate of substitution.

The emergence of the molecular clock hypothesis,[20–22] along with the neutral[23–25] and nearly neutral[26–28] theories of molecular evolution (reviewed by Ohta and Gillespie[29]) posited that a vast majority of nucleotide substitutions have neutral effect on fitness, which, in turn, indicate a constant substitution rate–for *all* sequences regardless of species–over time (like the ticking of a molecular clock). Although it is undeniable that neutral drift in sequence space must happen (given the finite size of all populations), the inaccuracies within the rates of molecular clocks (reviewed by Bromham[30]), together with the emergence of alternative molecular clock models (e.g., the episodic molecular clock[31,32]) and a better understanding of mutation rate change during speciation (reviewed below), we can be more certain that speciation events (and node formation in the tree) accounts for a substantial additional genome deviation.

One of the early pieces of evidence indicating that speciation events were concomitant with higher rates of molecular evolution was reported in 1990, when Mindell, Sites and Graur[2] found that a noticable increase in nucleotide substitution rates can be associated with speciation (or diversification) events in sceloporine lizards. Although this study dealt with lizards in particular, and the study *has* been contested,[33] an empirical preceedence was set in trying to determining the linkage between substitution rates and speciation. Today, evidence of linkage between molecular rates and speciation/diversification has been found within clades as diverse as angiosperms,[3] birds,[4,5] and confamilial sauropsids (birds and reptiles).[6] Importantly to the manuscript, a study of a large number of available phylogenies (unbiased by node density artifacts[34,35]) indicated direct correlations between node number (identical in form to the "node number" in the MS) and extent of sequence divergence from the last common ancestor[7–11] (for striking graphs, please refer to Fig. S1 in Webster *et al.*'s paper[7] and Fig. A3 in Venditti *et al.*'s paper[9]). This direction finally culminated in the finding that increased mutation rates during speciation events *explain* rate discrepancies in molecular clocks,[12] which was otherwise a scarcesly understood issue (with specific exceptions[36,37]). It is very interesting that, among a diverse group of phylogenetic trees, the discrepancies in the expected molecular clock rates have been explained merely by the additional effects of increasing substitution rates during cladogenesis (Fig. 4 in Pagel *et al.*'s report[12]).

From these studies, it appears incontrovertable that increased mutation rates during speciation events *do* significantly contribute to the rate of non-synonymous substitution that was previously predominantly thought to be dominated by neutral drift and phyletic gradualism.

S1.4 Oil escape is expected to happen predominantly during speciation events and less so during neutral drift.

As described in the previous section, the total number of substitutions (or total "deviation") of a sequence from its last common ancestor can be split into *(i)* the deviation caused by a time-dependent neutral drift (which is expected to be generally equal for all sequences today[2,12,23,24,29]) and a node-number-dependent burst of substitutions that are indicative of speciation events.[2–12] Pagel *et al.* provide an equation describing this cumulative drift[12] (which is a variation of an earlier formalism[2]), where the total amount of deviation (x) of a species from the common ancestor (placed at the root) in a phylogenetic tree is described as

$$x = n\beta + g \tag{1}$$

Here, n is the node number of the sequence (related to the number of speciation events in its lineage leading to the common ancestor), β is the rate of substitution during speciation one event, and g is the constant number of substitutions encountered by neutral drift (Mindell *et al.* describe g as time multiplied by a constant rate of divergence by neutral or "anagenic" drift[2]).

Given the previous section, the following inferences can be made:

- Constant neutral drift (g in Equation 1) acts on all genomes at approximately the same rate[2,12] (especially under the nearly neutral theory reviewed by Ohta and Gillespie[29]), and can not account for differences between species in the number of substitutions accumulated, i.e., *all sequences have diverged equally due to this phenomenon*.

- Neutral drift accounts for only those nucleotide substitutions that are "neutral" or "nearly neutral", which include synonyomous substitutions that display neutral fitness effects *and* a fraction of the non-synonymous mutations that display "nearly neutral" fitness effects.[23,24,29] This indicates that neutral drift would vastly account for mutations that are not under strong selective pressures, such as those mutations that maintain homology (or biochemistry) among protein sequences (as predicted by high log-odds scores for those residue substitutions in BLOSUM matrices[17]). As shown in Table S1, mutations that tend to change oil (FILV) or charge (ERDK) *content* within a sequence are significantly lower in log-odds probabilities than mutations that *maintain* sequence oil- and charge-composition, i.e., neutral drift manifested in non-speciation times of a genome (and the g component of Equation 1) is expected to have little effect on (oil and charge) composition change.

- Given the inability for neutral drift to explain the shift in a proteome's oil content, what else could explain such a phenomenon? An alternative explanation to neutral drift for the introduction of composition changing (and low-BLOSUM-score) substitutions into a sequence is by hitch-hiking of slightly deleterious mutations along with adaptive mutations (the genetic "draft" or hitch-kiking hypothesis[13–15]) during the burst of possibly adaptive mutations expected to accompany speciation events,[2–12] i.e., oil escape is expected to happen majorly under speciation events or creation of additional nodes in a species' lineage.

  The true phenotype of oil-composition-changing mutations–whether these mutations are functionally adaptive or non-adaptive and possibly deleterious–might indicate alternative mechanisms involving oil-composition-changing substitutions, however, those distinctions are not as important as the statement that these substitutions are *not* expected to neary neutral, and so, are not expected to happen during the Kimura (or Ohta) style neutral genetic drift. Also, as discussed in the next subsectionSection S1.1, a random drift in oil content is not capable of shifting the oil content of a proteome by unbiased random drift, and if fact, a bias (or "field" applied onto the random choces of substitution made, e.g., one that exists when starting at higher oil contents) is necessary to explain such an oil escape.

S2 The seed proteins used to produce Fig. 3A:

FASTA file of the seed protein used to produce Fig. 3A, left panel:

```
>d2fcwb1 g.12.1.1 (B:86-124) Ligand-binding domain of low-density lipoprotein receptor {Human (Homo sapiens) [TaxId: 9606]}
KTCSQAEFRCHDGKCISRQFVCDSDRDCLDGSDEASCPV
```

FASTA file of the seed protein used to produce Fig. 3A, center panel:

```
>d1jg1a_ c.66.1.7 (A:) Protein-L-isoaspartyl O-methyltransferase {Archaeon Pyrococcus furiosus [TaxId: 2261]}
EKELYEKWMRTVEMLKAEGIIRSKEVERAFLKYPRYLSVEDKYKKYAHIDEPLPIPAGQTVSAPHMVAIMLEIANLKPGMNILEVGTGSGWNAALISEIVKTDVYTIE
RIPELVEFAKRNLERAGVKNVHVILGDGSKGFPPKAPYDVIIVTAGAPKIPEPLIEQLKIGGKLIIPVGSYHLWQELLEVRKTKDGIKIKNHGGVAFVPLIGEYGWK
```

FASTA file of the seed protein used to produce Fig. 3A, right panel:

```
>d1qqea_ a.118.8.1 (A:) Vesicular transport protein sec17 {Baker’s yeast (Saccharomyces cerevisiae) [TaxId: 4932]}
ISDPVELLKRAEKKGVPSSGFMKLFSGSDSYKFEEAADLCVQAATIYRLRKELNLAGDSFLKAADYQKKAGNEDEAGNTYVEAYKCFKSGGNSVNAVDSLENAIQIFT
HRGQFRRGANFKFELGEILENDLHDYAKAIDCYELAGEWYAQDQSVALSNKCFIKCADLKALDGQYIEASDIYSKLIKSSMGNRLSQWSLKDYFLKKGLCQLAATDAV
AAARTLQEGQSEDPNFADSRESNFLKSLIDAVNEGDSEQLSEHCKEFDNFMRLDKWKITILNKIKESIQQQEDD
```

S3 Useful figures

A

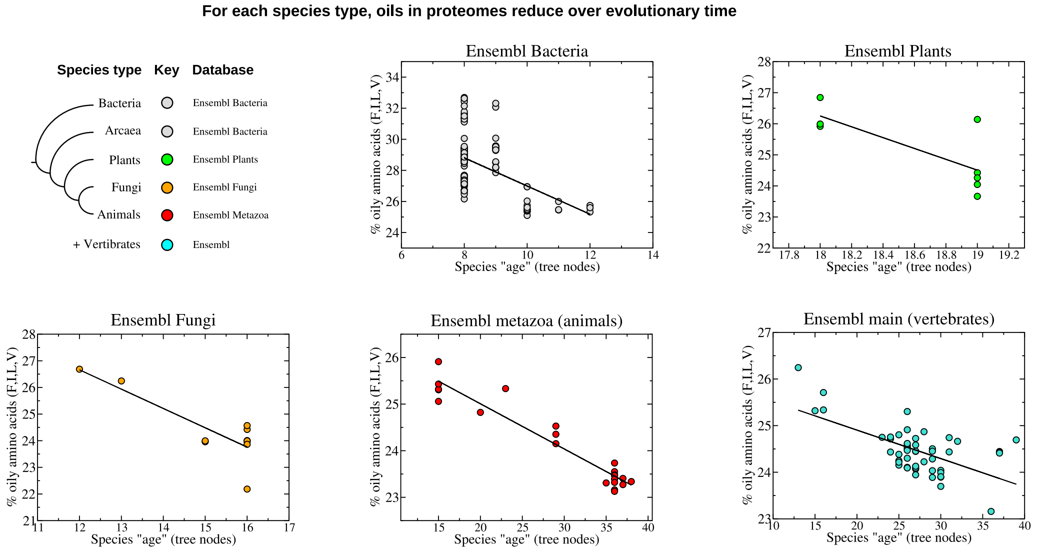

B

Ensemble database	# of unique proteomes	Spearman stats (r_s,p-val)	Pearson stats (r,p-val)	Kendal τ stats (r,p-val)
Plants	8	(-0.3810, 0.3518)	(-0.7595, 0.0288)	(-0.5368, 0.0630)
Metazoa	22	(-0.7818, 1.7e-5)	(-0.9589, 2.1e-12)	(-0.6940, 6.2e-6)
Main	51	(-0.4479, 9.8e-4)	(-0.6124, 1.8e-6)	(-0.3533, 2.5e-4)
Fungi	11	(-0.2841, 0.3972)	(-0.8387, 0.0013)	(-0.3990, 0.0875)
Bacteria	60	(-0.29328, 0.0230)	(-0.59938, 4.2e-7)	(-0.37432, 2.4e-5)
ALL	156	(-0.8464, 5.6e-44)	(-0.7743, 2.1e-32)	(-0.6882, 3.1e-37)

Figure S1: (**A**) is an expansion of the panels in Fig 1A that describe oil escape in even individual databases. Statistics of the correlations coefficients obtained by different methods are shown in (**B**). Each point represents proteomes belonging to distinct species (multiple datasets from the same species were merged).

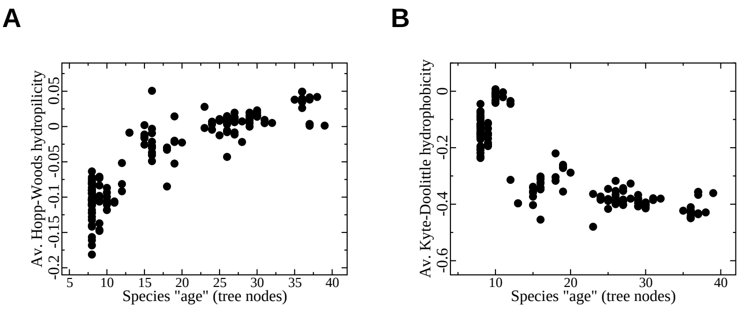

Figure S2: Reflecting the trends in the top four most hydrophobic residues (Fig. 2B), the proteome's average hydrophilicity increases over evolutionary time (**A**), while the proteome's average Kyte-Doolittle hydrophobicity decreases (**B**). In (**A**) the r and p-value for Spearman, Pearson and Kendal τ regression statistics are (0.8798, 2.6e-50), (0.8739, 7.7e-49) & (0.7003, 1.6e-37), respectively. For (**B**), those values respectively are (-0.8091, 1.9e-36), (-0.8266, 2.8e-39) & (-0.6197, 9.3e-30).

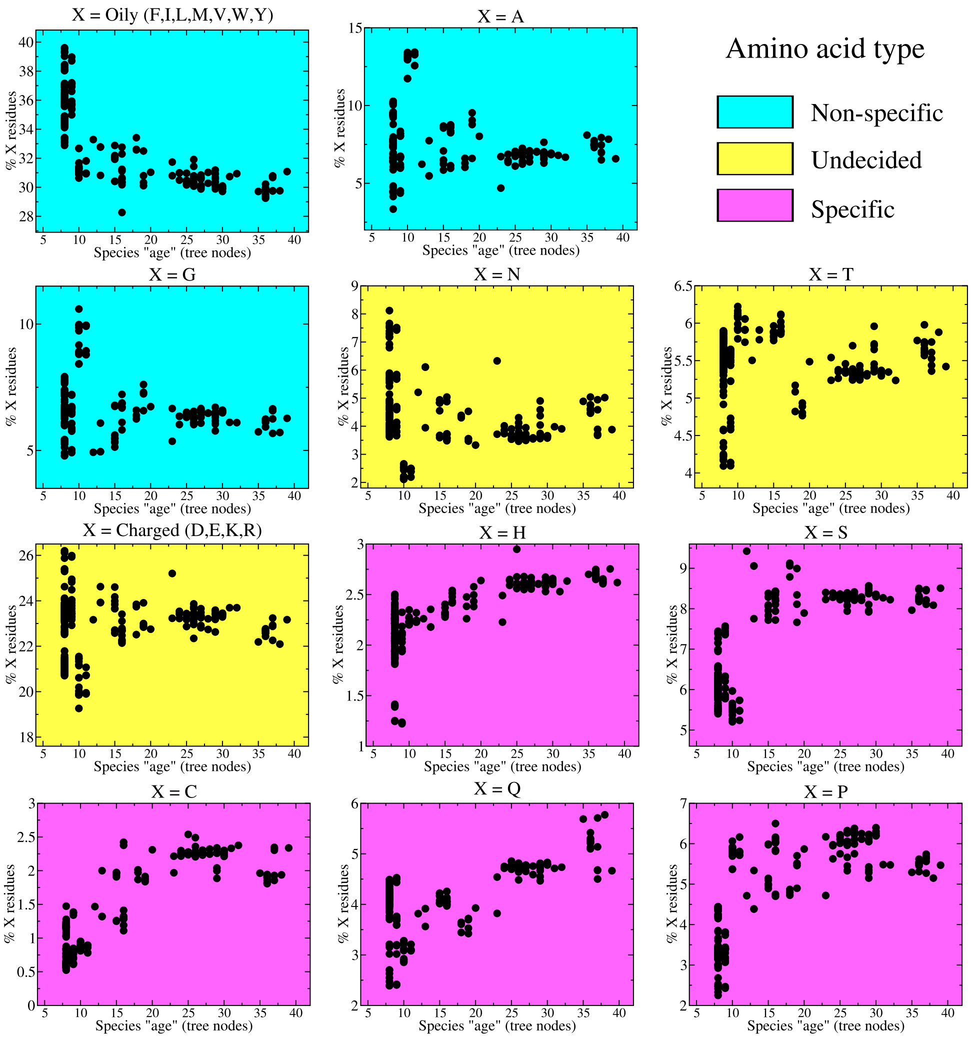

Figure S3: **Proteomes are increasing in "specificity" over evolutionary time.** An increase in the frequency of oily (non specific) residues, is mirrored by those residues that provide specificity to a protein (such as disulphide and hydrogen bonding polar residues), while those residues that have ambivalent features (such as charges, which may be used to maintain disorder, like in intrinsically disordered proteins, and order, like in proteins stabilized by salt bridges) show "ambivalent drift" over node space (which would indicate more niche-like constraints for these residues). These trends support the pluripotent mechanism (described in the text) as the originator of the protein repertoire.

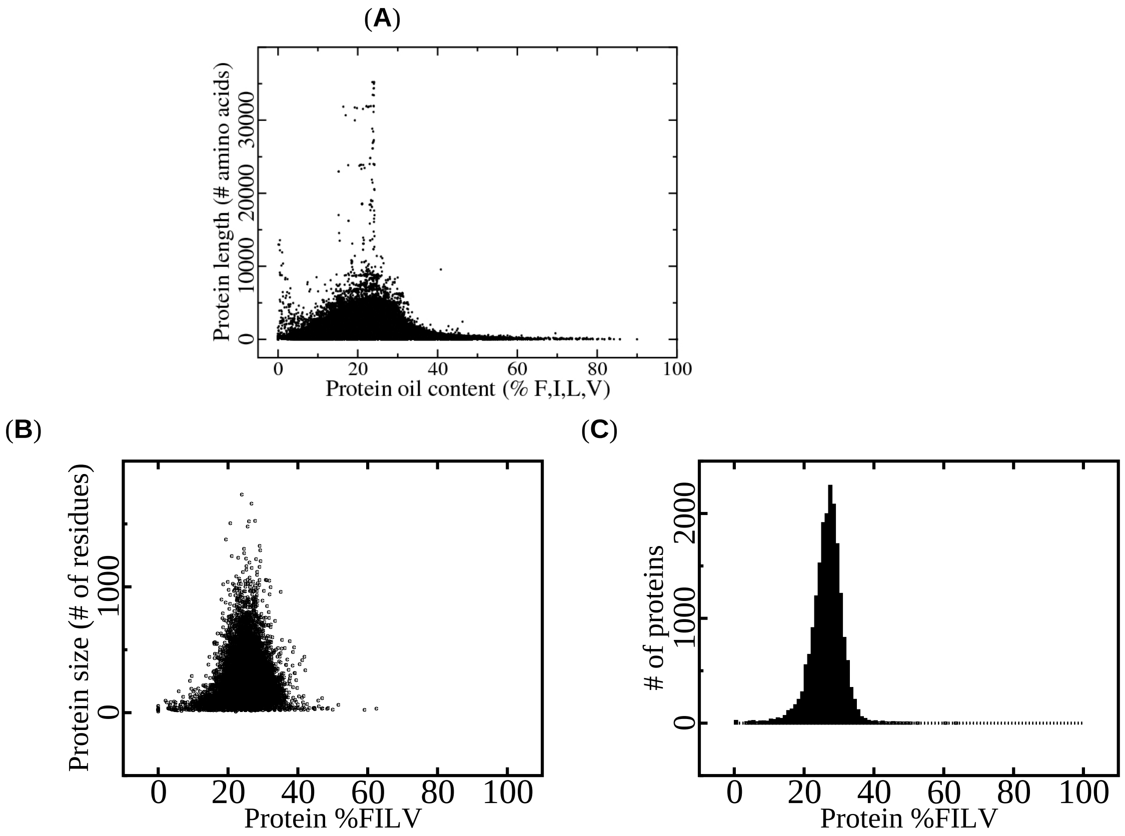

Figure S4: **Protein oil content is not a strongly controlled value.** A scatter plot of oil content vs. protein size in both the predicted proteomes (**A**) and a non-redundant set of the protein databank (**B**) indicates that an evolved protein's oil content depends little on its length (otherwise, one would expect the scatter plot to follow a curved trend). This indicates that, within the observed range, protein "oiliness" is not strongly controlled by protein design requirements, and so the drifts visible in Figs. 1 and 2 are functions of the proteome/protein's history rather than any specific protein design requirement. A histogram of protein oil content sourced from domains obtained from the SCOP database v1.75 is shown in (**C**) for reference.

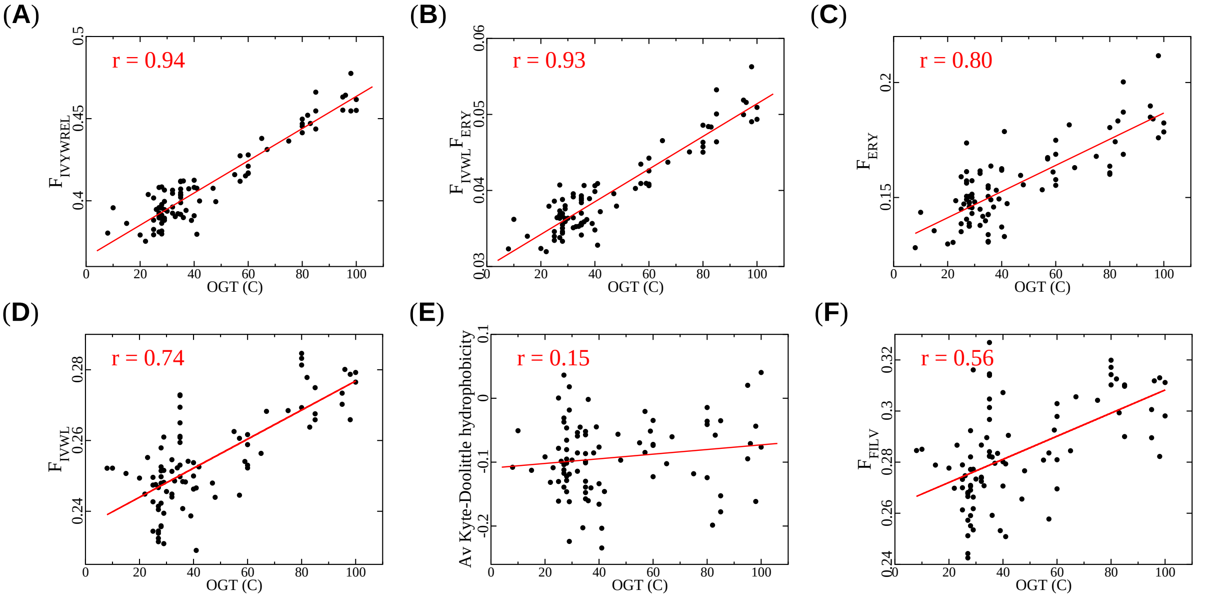

Figure S5: **Only full utilization of the IVYWREL combination provides excellent correlation with OGT in prokaryotes.** Although the summed fraction of amino acids IVYWREL (single amino acid codes used) in a proteome, $F_{IVYWREL}$, is an excellent predictor of a prokaryote's optimal growth temperature or OGT (**A**), both the individual summed fractions of relatively hydrophobic and non-hydrophobic components of IVYWREL (F_{IVWL} and F_{ERY}, respectively) are equally required to predict OGT, as indicated by the excellent correlation between OGT and $F_{IVWL} \cdot F_{ERY}$ (**B**). This excellent correlation is lost when considering only one of the two fractions (**C**,**D**), while the correlation is even less evident when looking at the proteome's average Kyte-Doolittle hydrophobicity (**E**) and summed fraction of the four oiliest amino acids rated by the Kyte-Doolittle scale (**F**). The prokaryotic proteomes and their corresponding OGTs used are identical to those described previously.[38]

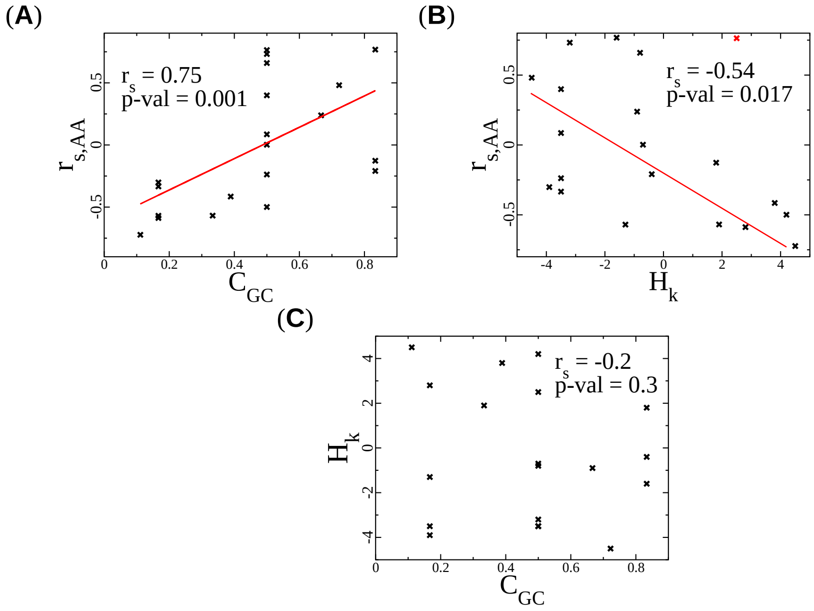

Figure S6: Both the amino acid's average nucleotide GC fraction per codon or C_{GC} (**A**) *and* Kyte-Doolittle hydrophobicity or H_k (**B**) correlate well with the extent of each amino acid's evolutionary "drift" over node space (assessed by the Spearman correlation number $r_{s,AA}$ between the fraction of a single type of amino acid F_{AA} in a proteome and species node number, which are explicitly shown in Fig. S8). Note that, in panel **B**, Cysteine, marked as a red "x", was discounted when calculating r_s, due to it's non-canonical property of possessing high hydrophobicity *and* high interaction specificity). However, C_{GC} and H_k are themselves *not* even negligibly correlated (given the high probability, p-val$=0.3$, that the scatter plot in **C** is random). This indicates that there are two separate constrains/drifts at play simultaneously: one at the genome/nucleotide level (GC drift) and one at the proteome/amino acid level (which is the "the oil escape" discussed in the main text). Actual plots of individual amino acid drifts are displayed in Fig. S8.

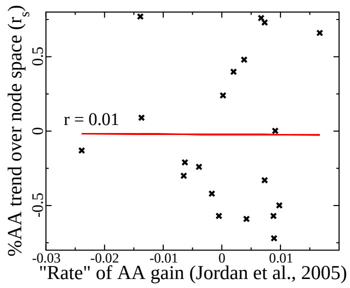

Figure S7: The extent of each amino acid's evolutionary "drift" over node space (assessed by the Spearman correlation number $r_{s,AA}$) does not correlate with predicted rates of increase/decrease of an amino acid per substitution per protein site [obtained from Table 3 in[39]]. Actual plots of individual amino acid drifts are displayed in Fig. S8.

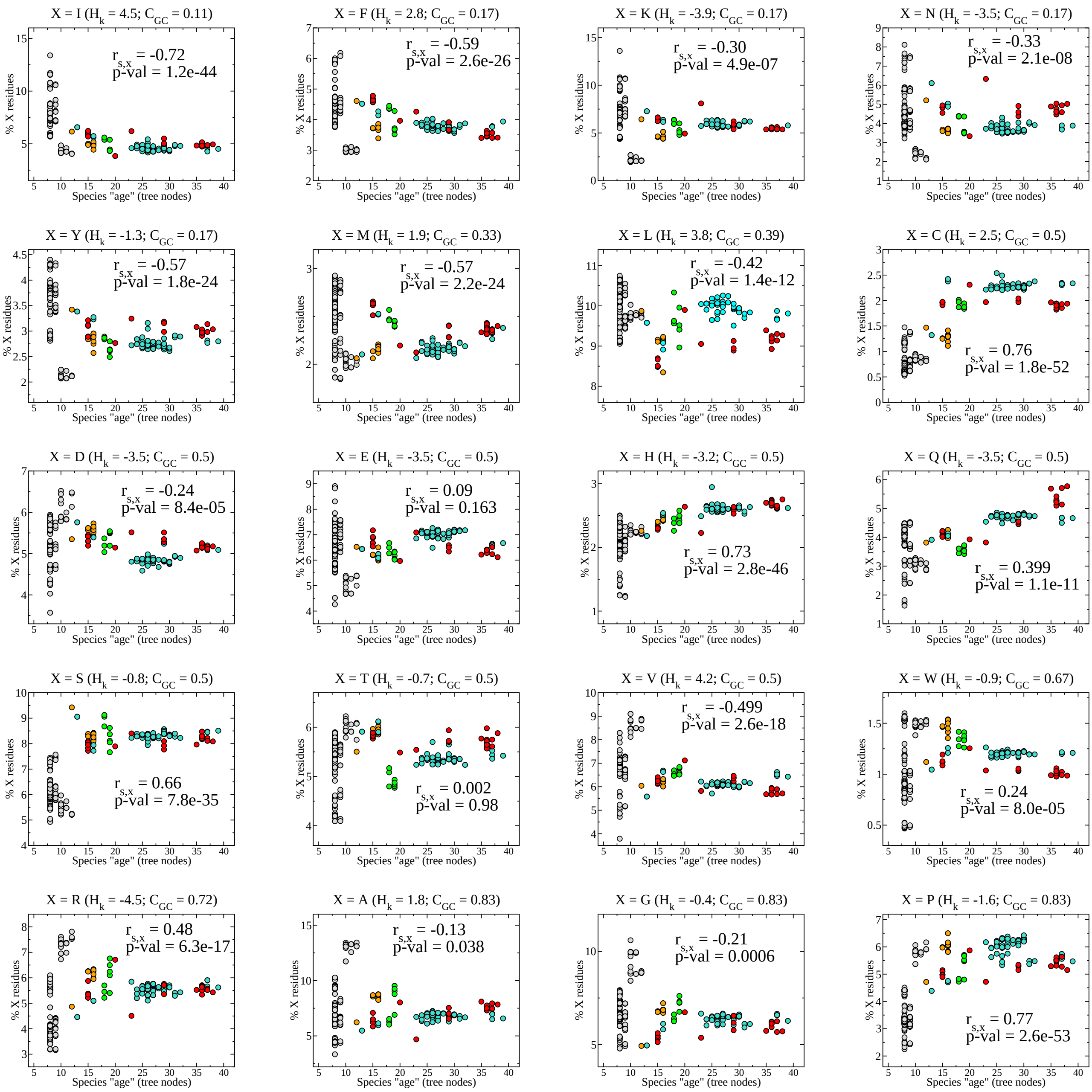

Figure S8: Single amino acid abundances in a proteome (or $100 \times F_{AA}$) plotted over species nodespace. Also shown for each amino acid are the following values that are used in Fig. S6: the average nucleotide GC fraction in a codon (C_{GC}), Kyte-Doolittle hydrophobicity (H_k), Spearman rank correlation ($r_{s,AA}$) and its corresponding p-value (p-val).

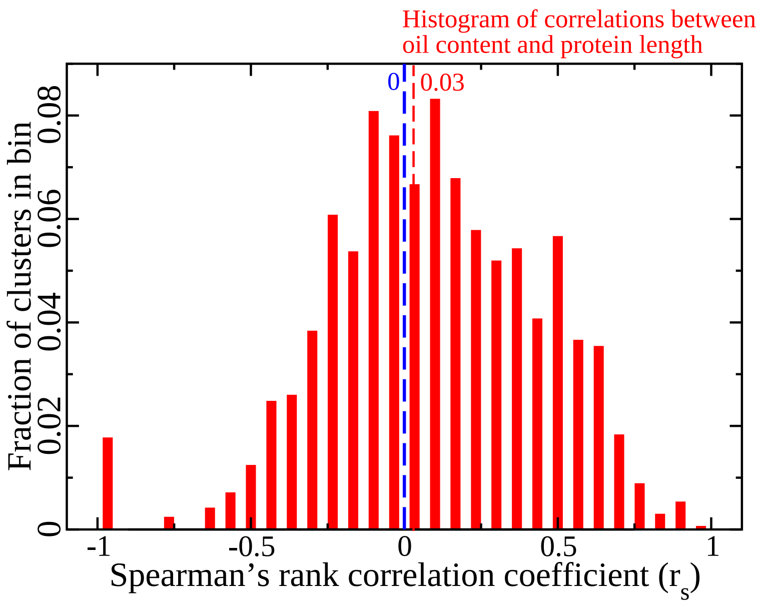

Figure S9: **Protein length within size-homogenized clusters does not correlate with oil content as described by the gaussian distribution of Spearman correlations of length vs. oil content within clusters.** For every size-homogenized cluster showing a statistically significant change in oil content (1697 in total), we calculated the Spearman correlation coefficients (r_s) between a protein's oil content versus its length, which is plotted in histogram form. The mean r_s in this study lies very close to zero (at $r_s \sim 0.03$), indicating a relatively unbiased dependence of oil content on protein length within size-homogenized homologous clusters. The broad distribution of these r_s's exist due to the small average size of proteins per cluster. These results indicate that oil escape within globular protein domains was not driven primarily by the addition of loops and intrinsically disordered regions within globular proteins (which would cause the increase in protein length).

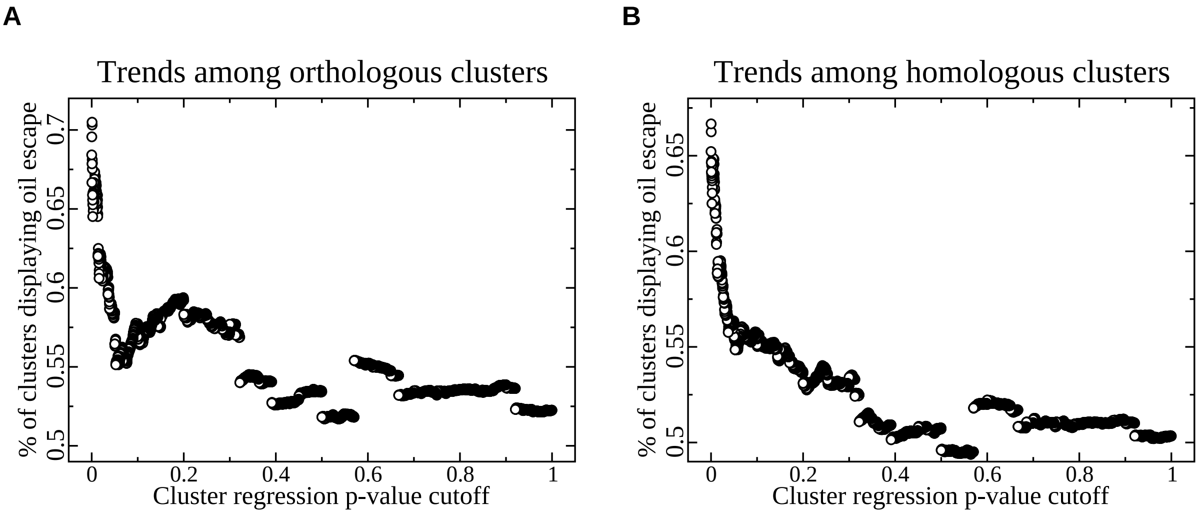

Figure S10: **Among both groups of orthologous proteins (A) and homologous (B) available in the COG database, oil escape is evident.** The single protein studies of Fig. 3 were collected my obtaining the most similar protein from each of the proteomes. In doing so, each cluster is a collection of a orthologs (if we find one for the seed protein) and paralogs (if we do not find an ortholog). However, to many, this study is flawed, as paralogs (that may or may not be directly related to the seed protein) are added to the cluster, thereby reducing the validity of the study. In response, we have performed two new studies using the most recent COG database (each cluster in this database corresponds with a Cluster of Orthologous protein groups ranging through 66 complete genomes[40]). From the database, we performed our regression analys on both exclusively orthologous protein clusters (**A**) and homologous clusters (**B**). The available clusters unfortunately, due to sampling problems (because of smaller dataset size and a shorter range of node numbers as only lower-eukaryotes have been included in the study), often result in statistically irrelevant correlations. However, when we shift the threshold for statistical relavance, we find that more and more number of clusters display oil escape, which strictly tend to favor oil escape as we pass the significance threshold of p-value ≤ 0.05. Also, interestingly, the statistically significant oil escapes actually increase in percent as one goes from a mix of homologous (orthologous and paralogous) clusters to exclusively orthologous clusters, which only reasserts the claims regarding oil escape among clusters of related proteins.

Details:

The tree of life comprises a collapsed, pruned, tree obtained from the iToL website. The newick format of this tree is as follows:

((((Methanopyrus_kandleri, (Thermoplasma_volcanium, Thermoplasma_acidophilum)Thermoplasma, Archaeoglobus_fulgidus, Methanocaldococcus_jannaschii, Halobacterium_sp., Methanothermobacter_thermautotrophicus, (Pyrococcus_horikoshii, Pyrococcus_abyssi)Pyrococcus, Methanosarcina_acetivorans)Euryarchaeota, (Pyrobaculum_aerophilum, Sulfolobus_solfataricus, Aeropyrum_pernix)Thermoprotei)Archaea, (Deinococcus_radiodurans, (Clostridium_acetobutylicum, (((Bacillus_subtilis, Bacillus_halodurans)Bacillus, Staphylococcus_aureus, Listeria_innocua)Bacillales, (Lactococcus_lactis, (Streptococcus_pneumoniae, Streptococcus_pyogenes)Streptococcus)Streptococcaceae)Bacilli)Firmicutes, Thermotoga_maritima, (Chlamydophila_pneumoniae, Chlamydia_trachomatis)Chlamydiaceae, (((Haemophilus_influenzae, Pasteurella_multocida)Pasteurellaceae, Vibrio_cholerae, Pseudomonas_aeruginosa, (Buchnera_sp., Escherichia_coli, Yersinia_pestis, Salmonella_enterica)Enterobacteriaceae, Xylella_fastidiosa)Gammaproteobacteria, ((Brucella_melitensis, (Sinorhizobium_meliloti, Agrobacterium_tumefaciens)Rhizobiaceae, Mesorhizobium_loti)Rhizobiales, Caulobacter_vibrioides, (Rickettsia_prowazekii, Rickettsia_conorii)Rickettsia)Alphaproteobacteria, (Campylobacter_jejuni, Helicobacter_pylori)Campylobacterales, (Neisseria_meningitidis, Ralstonia_solanacearum)Betaproteobacteria)Proteobacteria, (Ureaplasma_urealyticum, (Mycoplasma_pulmonis, Mycoplasma_genitalium, Mycoplasma_pneumoniae)Mycoplasma)Mycoplasmataceae, (Synechocystis, Nostoc_sp.)Cyanobacteria, Aquifex_aeolicus, (Borrelia_burgdorferi, Treponema_pallidum)Spirochaetaceae, (Corynebacterium_glutamicum, (Mycobacterium_leprae, Mycobacterium_tuberculosis)Mycobacterium)Corynebacterineae, Fusobacterium_nucleatum)Bacteria, (Encephalitozoon_cuniculi, (Saccharomyces_cerevisiae, Schizosaccharomyces_pombe)Ascomycota)Fungi)cellular_organisms)

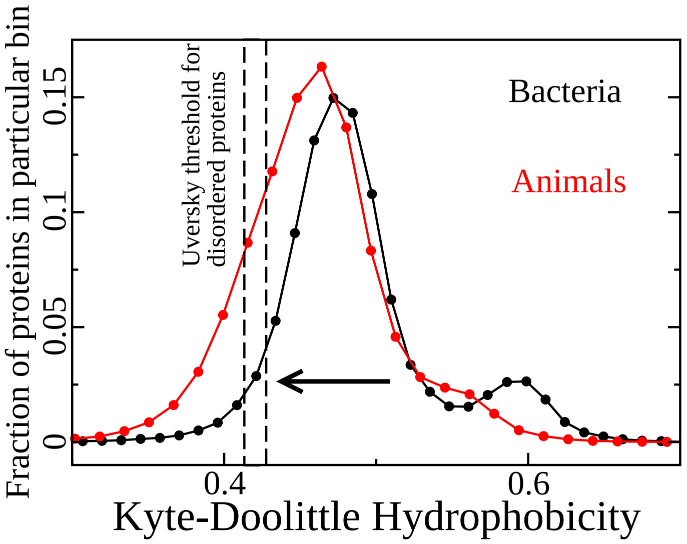

Figure S11: **Oil escape is described by a shift in the near-gaussian distribution of oil content per protein, which precludes theories favoring the preferential adaptation of IDPs in complex organisms.** The figure above provides two histograms, that describes the distribution of protein oil content (this time using the Kyte-Doolittle scheme) among all proteins found in proteomes belonging to all animals (red curve) and bacteria (black curve) described in the SI. It is interesting to note that the two distributions are generally Gaussian-like, and, as discussed in the MS and SI, we expect that the distribution described by the bacterial aggregate would most resemble the last common universal ancestor's oil content, and the red curve represents newer proteomes that emerged only later; this picture can be described by the shift of a unimodal (gaussian) distribution of protein oil contents to lower mean oil contents, where the gaussian distribution slides to the left in the picture. It is important to note that Uversky (Protein Sci. 2002, 11(4):739-56) has shown that a strong divide between IDPs and globular/folded proteins is dictated primarily by a threshold hydrophobicity and secondarily (to a less important degree) by the protein's mean net charge per amino acid. The two vertical, dashed lines describe the threshold for proteins ranging in 10 varying charge configurations (which would involve a large number of proteins we know). The band delineated by the two lines indicates a general sequence-composition-threshold describing the transition between globular proteins and IDPs. If IDPs were to be enriched in later proteomes due to adaptation, then one would expect to find a more aggressive recruitment/invention of IDPs in the proteome, from which we would predict a second hump in the protein hydrophobicity histogram in complex organisms (red). However, all we see in animal protomes, is a unimodal (single hump) oil-distribution whose tail drifts into the Uversky threshold, thereby putatively allowing for the chance encountering of IDPs in the first place. Given the lack of a new hump in the "Uversky zone" (left of the dashed lines) which would indicate the active enrichment of IDPs in complex organisms, we can infer that Uversky-like IDPs didn't cause, but emerged from the Gaussian shift in oil content over evolutionary time. In conclusion, oil escape since the emergence of the LUCA explains the emergence of IDPs, and there is no reason to expect that any adaptive force drove the emergence of IDPs.

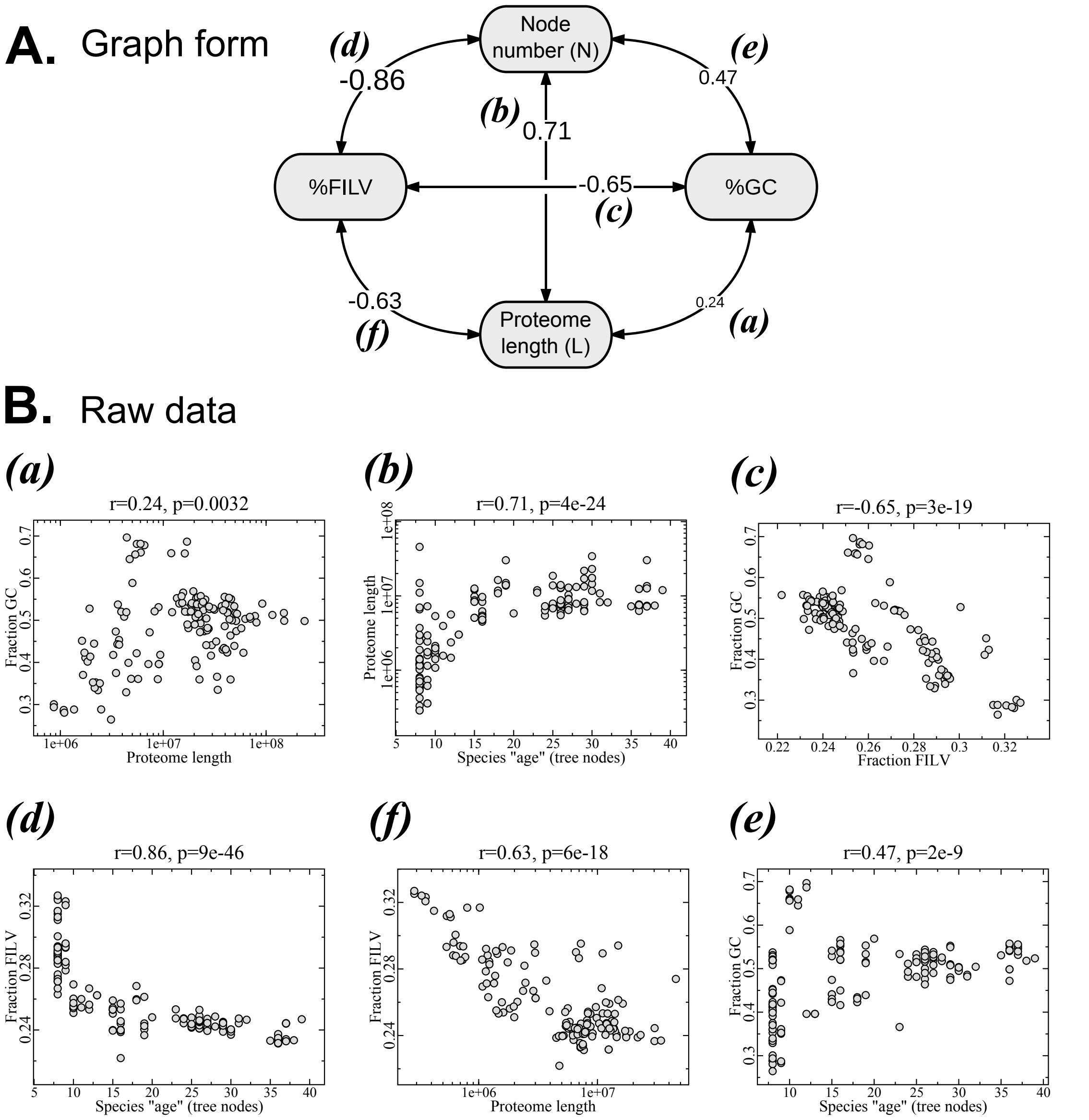

Figure S12: **The complete correlation graph (A) along with the individual relationships (B).** $\%FILV$ vs node number displays the strongest correlation between the four relationships: $\%FILV$, $\%GC$ (obtained from ensemble cDNA transcripts), node number, and proteome length (L). The complete correlation network indicates that while GC content is correlated with node number, only three correlations–(*i*), (*ii*) and (*iv*)–may be sufficient in explaining all other correlations. Also, it is unlikely that the weak relationship between GC content and node number will be able to cause the relationship between FILV and node number, i.e., the relationship between GC content and node number is likely either caused by the coupling between FILV and GC (*ii*) or by unknown and independent constraints.

(**A**) (**B**)

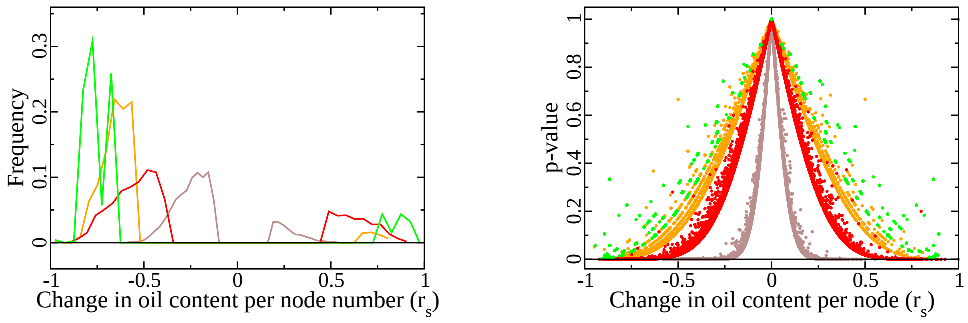

(**C**)

Ensemble database	Statistically significant cluster C_t ($p \leq .05$)	Statistically significant clusters with $r_s \leq 0$ (C_o)	Percent of clusters showing oil escape ($100 \times C_o/C_t$)
Bacteria	2980	2506	84.094%
Metazoa	1719	1233	71.728%
Fungi	758	713	94.063%
Plants	963	832	86.397%

Figure S13: Similar to the analysis of homologous protein clusters within the cumulative database (Figs. 3B,C), analogous studies (the normalized distribution of the spearman correlation r_s within clusters that showed p-val < 0.05: **A**; **B** describes the scatter relationship between r_s and p-val for each cluster) where clusters were limited to one of four databases–Ensemble bacteria (grey), plants (green), metazoa (red), and fungi (orange)–resulted in qualitatively identical inferences (statistics in **C**): oil escape occurs dominantly even the individual databases. Clusters were only used if the change in oil content per node number (r_s) is statistically significant (p-val < 0.05).

(A) (B)

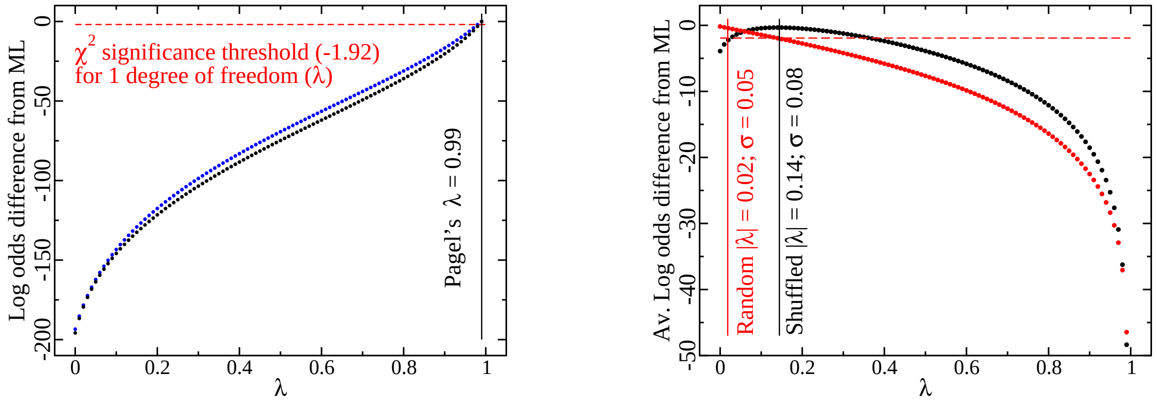

(C)

Database	Lambda	α	σ^2
Bacteria	1.00	29.257	0.309
Metazoa	0.98	25.092	0.031
Metazoa	1.00	25.500	0.0176
Fungi	1.00	25.395	0.193
Plants	0.97	25.356	0.097
Combined	0.99	28.149	0.165

Figure S14: **Finding Pagel's maximum likelyhood λ for the original ToL (A), and the randomized ToL's (B).** Pagel's λ metric[41,42] is used to distinguish between whether, given a species tree, and a species trait (assigned for the leaves in the tree) is independent of phylogenetic relationships ($\lambda = 0$) or dependent on the species' genetic shared history ($\lambda = 1$). Using the same methedology described in Figure 1 of Freckleton *et. al*,[42] we find that the maximum likelyhood (ML) probability of λ in our combined database (**A**), *and* our individual databases (**C**) indicate very high estimated λ's (≥ 0.97). The $\lambda = 0.99$ estimate for the original tree, with branch lengths of unit length (**A**; black circles), are also obtained for a "neutral drift" tree, where branch lengths were modified to ensure that all species-to-root lengths are equal (**A**; red circles). Also, the critical value of the log-likelihood ratio test (red dashed line; p-val$= 0.05$) indicates that the value of λ is significantly different from 0 (i.e., a non-phylogenetic explanation for the character trait, oil escape, is highly unlikely). As a control, we studied 1000 original trees whose species character trait values were *(i)* shuffled and *(ii)* randomized with a standard normal probability distribution ($\mu = 1$,$\sigma^2 = 1$). Panel (**B**) describes the search for Pagel's ML λ's for the shuffled (black) and randomized (red) trees, which resulted in estimated λ values of 0.14 ± 0.08 and 0.08 ± 0.05, respectively.

Calculating λ: Each branch in the iToL/NCBI tree used (See Section S6.2) is set to 1, which means that the "operational time" of this tree is in node numbers. A variance-covariance matrix **V** of the tree (where diagonal elements $\mathbf{V}_{i,i}$ were set to the node number of species i, and the off diagonal elements $\mathbf{V}_{i,j}$ are the shared history (in node numbers) between the two species i and j. $\mathbf{V}(\lambda)$ is a modification of **V** by an off diagonal multiplier λ (normall $\in$ $[0, ..., 1]$). At each value of λ, we calculate the likelyhood of $p(\lambda, \mathbf{y})$ (Equation 4 in Freckleton et al.[42]), which will end up giving us the most likely (ML) λ value. The $x - axis$ in the graphs indicate the difference $-\left[ln\left(p(\lambda_0, \mathbf{y})/p(\lambda_1, \mathbf{y})\right)\right]$, where λ_0 is the maximum likelihood λ, and $\lambda_1 \neq \lambda_0$ is the null hypothesis. Any difference ($-\left[ln\left(p(\lambda_0, \mathbf{y})/p(\lambda_1, \mathbf{y})\right)\right]$) that is lower than the χ^2 significance cutoff (-0.92 or the red dashed line, which is obtained from precompiled tables setting p-val$= 0.05$ with one degree of freedom) indicate rejected values for λ.

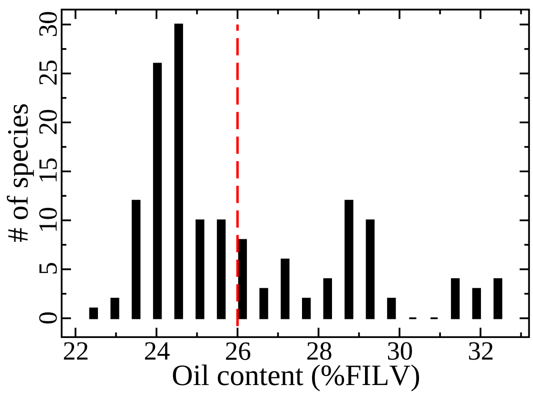

Figure S15: **Histogram distribution of proteome oil contents (number of bins: 20)**. Given the non-gaussian distribution around 26%FILV (red dashed line), the diffusion of evolving oil contents from an ancestral value of 26% is unlikely.

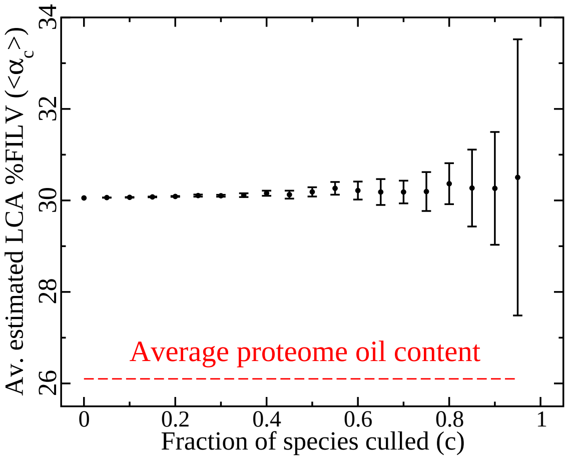

Figure S16: **Oil escape is robust to data culling.** For each culling fraction (C), we obtained 100 species sets each amounting to a subset of the total number of species in our database. From each set, utilizing the original tree and the subset of oil contents per species, we obtained using a generalized least squares method[43] the estimated LCA's oil content. For each C, the resulting 100 estimated ancestral oil contents were averaged and displayed above (with error bars representing variance). It appears as though our last common ancestor's oil content is consistently predicted on average to be similar in value (between 30 and 30.5). While the mean is steady across testing parameters, the variance (σ^2) of the estimation per C increases, but is remarkably low even with 70% of the species culled. This indicates that while the set of sequenced species in relatively low, the LCA's oil content is consistently predicted to be higher than average (26.1). This study was repeated independently with no difference in inference.

METHOD:

Set up for estimating the ancestral oil content: Each branch in the iToL/NCBI tree used (See Section S6.2) is set to 1, which means that the "operational time" of this tree is in node numbers (an alternative tree was also used to model neutral drift). An $n \times n$ variance-covariance matrix $\mathbf{V}$ of the tree (where diagonal elements $\mathbf{V}_{i,i}$ were set to the node number of species i, and the off diagonal elements $\mathbf{V}_{i,j}$ are the shared history (in node numbers) between the two species i and j in our ToL). Let $\mathbf{Y}_i$ be the $n \times 1$ (column) vector of character states (oil contents), i.e., for any species i, $\mathbf{Y}_i$ is $\%FILV_i$. Finally, let $\mathbf{T}$ be a $n \times 2$ matrix whose first column elements all equal 1 and the second column elements depicts the species node number (i.e., $\mathbf{T}_{i,1} = 1$, $\mathbf{T}_{i,2} = i$'s node number).

Estimating the ancestral oil content: Then, the model of evolution[43] for species i is $\mathbf{Y}_i = \alpha + \beta \mathbf{T}_{i,2} + \epsilon_i$, where α is the character state of the ancestor at operational time 0 (i.e., the LCA), β is the estimated rate of change of the character state per operational time unit (e.g., node number), and ϵ_i is the random error.[43] From a generalized least squares method,[43] we can estimate both α and β by solving for
$(\beta, \alpha) = (\mathbf{T}^T \mathbf{V}^{-1} \mathbf{T})^{-1} (\mathbf{T}^T \mathbf{V}^{-1} \mathbf{Y})$.

S4 Statistics of the asymptotic nature of oil escape.

We use the following asymptotic form to fit the scatter plot (oil escape) described in Fig. 2B:

$$\%FILV = \phi_1 + (\phi_2 - \phi_1)\, e^{-e^{(\phi_3)} ln\,(N)} \tag{2}$$

Here, N is its node number, ϕ_1 is the asymptote, ϕ_2 is the ordinate-intersect and ϕ_3 is the rate constant of the trend.

Keeping all ϕ's variable, and fitting to the data in Fig. 2B, we obtained the following values: $r = 0.879, \phi_1 = 23.91\%, \phi_2 = 883.6, \phi_3 = 0.89$. Also, **constraining** $\phi_1 = \%FILV^C$ still results in the following values: $r = 0.877, \phi_2 = 301.49, \phi_3 = 0.62$), which is higher than the corresponding monotonicity-based Spearman coefficient r_s of ~ 0.846.

S5 The proteomes analyzed

The “proteomes” analyzed were annotated from genomes obtained from the Ensembl databases.[44] All species listed below (and in Table S2) were used to create Fig. 2A,B, Fig. 3 and Fig. S4, while only the italicized ones (those with first occurrence data in brackets available from pbdb.org) were used to calculate Fig. 2C.

Ensembl plants; release-4 [# of species: 7; genomes: 8]: Arabidopsis lyrata; Arabidopsis thaliana; Brachypodium distachyon; Oryza sativa; *Populus trichocarpa* (89.300 Ma); Sorghum bicolor; *Vitis vinifera* (99.600 Ma); **Ensembl fungi; release-4** [# of species: 11; genomes: 11]: Aspergillus clavatus; Aspergillus flavus; Aspergillus fumigatus; Aspergillus niger; Aspergillus oryzae; Aspergillus terreus; Emericella nidulans; Neosartorya fischeri; Neurospora crassa; Saccharomyces cerevisiae; Schizosaccharomyces pombe; **Ensembl metazoa; release-4** [# of species: 22; genomes: 22]: *Aedes aegypti* (37.200 Ma); *Anopheles gambiae* (23.030 Ma); Caenorhabditis brenneri; Caenorhabditis briggsae; Caenorhabditis elegans; Caenorhabditis japonica; Caenorhabditis remanei; *Culex quinquefasciatus* (37.200 Ma); *Drosophila ananassae* (23.030 Ma); *Drosophila erecta* (23.030 Ma); *Drosophila grimshawi* (23.030 Ma); *Drosophila melanogaster* (23.030 Ma); *Drosophila mojavensis* (23.030 Ma); *Drosophila persimilis* (23.030 Ma); *Drosophila pseudoobscura* (23.030 Ma); *Drosophila sechellia* (23.030 Ma); *Drosophila simulans* (23.030 Ma); *Drosophila virilis* (23.030 Ma); *Drosophila willistoni* (23.030 Ma); *Drosophila yakuba* (23.030 Ma); Ixodes scapularis; Pediculus humanus; **Ensembl main; release-57** [# of species: 51; genomes: 51]: *Anolis carolinensis* (20.430 Ma); *Bos taurus* (3.600 Ma); Caenorhabditis elegans; Callithrix jacchus; *Canis familiaris* (5.330 Ma); *Cavia porcellus* (1.810 Ma); *Choloepus hoffmanni* (0.011 Ma); Ciona intestinalis; Ciona savignyi; Danio rerio; *Dasypus novemcinctus* (1.810 Ma); *Dipodomys ordii* (1.810 Ma); *Drosophila melanogaster* (23.030 Ma); *Echinops telfairi* (0.011 Ma); *Equus caballus* (7.250 Ma); *Erinaceus europaeus* (11.610 Ma); *Felis catus* (20.430 Ma); *Gallus gallus* (13.650 Ma); *Gasterosteus aculeatus* (1.810 Ma); Gorilla gorilla; *Homo sapiens* (7.250 Ma); *Lama pacos* (1.810 Ma); *Loxodonta africana* (11.610 Ma); *Macaca mulatta* (11.610 Ma); *Macropus eugenii* (5.330 Ma); *Meleagris gallopavo* (5.330 Ma); *Microcebus murinus* (0.011 Ma); *Monodelphis domestica* (1.810 Ma); *Mus musculus* (7.250 Ma); *Myotis lucifugus* (33.900 Ma); *Ochotona princeps* (11.610 Ma); *Ornithorhynchus anatinus* (0.011 Ma); *Oryctolagus cuniculus* (3.600 Ma); Oryzias latipes; *Otolemur garnettii* (1.810 Ma); Pan troglodytes; *Pongo pygmaeus* (1.810 Ma); *Procavia capensis* (7.250 Ma); *Pteropus vampyrus* (0.011 Ma); *Rattus norvegicus* (5.330 Ma); Saccharomyces cerevisiae; *Sorex araneus* (15.970 Ma); *Spermophilus tridecemlineatus* (13.650 Ma); *Sus scrofa* (13.650 Ma); Taeniopygia guttata; Takifugu rubripes; Tarsius syrichta; *Tetraodon nigroviridis* (7.250 Ma); Tupaia belangeri; *Tursiops truncatus* (20.430 Ma); *Xenopus tropicalis* (83.500 Ma); **Ensembl bacteria; release-4** [# of species: 60; genomes: 176]: **Bacillus:** B amyloliquefaciens; B anthracis; B cereus; B clausii; B halodurans; B licheniformis; B pumilus; B subtilis; B thuringiensis; B weihenstephanensis; **Borrelia:** B afzelii; B burgdorferi; B duttonii; B garinii; B hermsii; B recurrentis; B turicatae; **Escherichia:** E coli; E fergusonii; **Mycobacterium:** M abscessus; M avium; M bovis; M gilvum; M leprae; M marinum; M paratuberculosis; M smegmatis; M sp; M tuberculosis; M ulcerans; M vanbaalenii; **Neisseria:** N gonorrhoeae; N meningitidis; **Pyrococcus:** P abyssi; P furiosus; P horikoshii; **Shigella:** S boydii; S dysenteriae; S flexneri; S sonnei; **Staphylococcus:** S aureus; S carnosus; S epidermidis; S haemolyticus; S saprophyticus; **Streptococcus:** S agalactiae; S dysgalactiae; S equi; S gordonii; S mutans; S pneumoniae; S pyogenes; S sanguinis; S suis; S thermophilus; S uberis; **Wolbachia:** W pipientis; W sp; **Others:** Buchnera aphidicola; Thermococcus kodakarensis.

Table S2: Ensembl genome species used in proteome analysis.

#	Species	Node #	FILV	hydrophobicity	hydrophilicity
**Ensembl main**					
1	Anolis carolinensis	26	24.911121921	-37.9224731411	-0.560508853123
2	Bos taurus	28	24.8695814744	-32.6894842967	-2.189837377
3	Caenorhabditis elegans	15	25.3194997509	-35.5108639787	-1.5426787175
4	Callithrix jacchus	29	23.8884369539	-40.7231414012	1.96419299624
5	Canis familiaris	27	24.583991022	-35.2867980839	-0.833873186611
6	Cavia porcellus	27	24.723829181	-34.2894022262	-1.13370593864
7	Choloepus hoffmanni	24	24.7554459523	-38.4512700898	0.31720135025
8	Ciona intestinalis	16	25.3365449842	-34.2299844742	-2.72998068218
9	Ciona savignyi	16	25.7095193918	-30.201364149	-4.89951866011
10	Danio rerio	29	24.503101874	-38.6693342081	1.18259983786
11	Dasypus novemcinctus	24	24.4330223641	-38.1913763293	0.687915187915
12	Dipodomys ordii	28	24.2240804182	-38.0320355969	0.728464481919
13	Drosophila melanogaster	36	23.159014235	-44.93002556	4.92973342304
14	Echinops telfairi	25	24.1525410468	-38.1842395841	0.953405354435
15	Equus caballus	26	24.5712428821	-38.6189454203	0.907697729072

Continued on next page

Table S2 – Ensembl species used in proteome analysis (continued)

#	Species	Node #	FILV	hydrophobicity	hydrophilicity
16	Erinaceus europaeus	26	24.4693277214	-37.7131916563	0.503128308006
17	Felis catus	27	24.0689332433	-38.742776961	1.19833648994
18	Gallus gallus	31	24.4355771038	-38.5150591055	0.936768904909
19	Gasterosteus aculeatus	37	24.4112204745	-35.6972849699	0.359596745323
20	Gorilla gorilla	30	23.9035228754	-40.1372309751	1.59182196113
21	Homo sapiens	30	23.6964160418	-41.4699769912	2.29085623766
22	Loxodonta africana	25	24.8028651582	-34.5788163968	-1.24563707322
23	Macaca mulatta	30	23.9922949938	-40.3049329081	1.67539532823
24	Macropus eugenii	24	24.7163026725	-37.2749993598	-0.45170446957
25	Meleagris gallopavo	31	24.7414987456	-37.8947459178	0.481045670043
26	Microcebus murinus	27	24.0916996464	-38.7760286046	1.19442080717
27	Monodelphis domestica	25	24.2473052742	-41.6562130423	0.805368641829
28	Mus musculus	29	24.0321952599	-39.3714201592	1.50666514033
29	Myotis lucifugus	26	24.0920547593	-39.3157962755	1.47620280951
30	Ochotona princeps	26	24.1048232818	-37.8446231597	1.03309945249
31	Ornithorhynchus anatinus	23	24.7482751061	-36.3727526204	-0.209309757986
32	Oryctolagus cuniculus	26	24.3000971515	-36.6258167919	0.301933114886
33	Oryzias latipes	39	24.6934165372	-36.0665477443	0.128224550015
34	Otolemur garnettii	27	24.4471734116	-38.3705222972	0.617076618967
35	Pan troglodytes	30	23.8944048571	-40.8676552352	1.98758067598
36	Pongo pygmaeus	30	24.0402683862	-39.6970157197	1.3852391338
37	Procavia capensis	25	24.2151368758	-38.2114295718	0.984775322929
38	Pteropus vampyrus	27	24.127981257	-38.7617422245	1.2708565967
39	Rattus norvegicus	29	24.2843086452	-38.0597885541	0.863121526983
40	Saccharomyces cerevisiae	13	26.2440278199	-39.6793954764	-0.869751279095
41	Sorex araneus	26	24.5089856732	-38.0386057345	0.790959255291
42	Spermophilus tridecemlineatus	29	24.4514213736	-38.2035364668	0.46397334998
43	Sus scrofa	26	24.546984679	-35.3082939549	-0.794265615244
44	Taeniopygia guttata	32	24.6630333572	-38.01887545	0.506469144419
45	Takifugu rubripes	37	24.4446391185	-36.6941745333	0.110838545363
46	Tarsius syrichta	27	24.452207868	-40.2918262635	1.32081597686
47	Tetraodon nigroviridis	37	24.4269330073	-35.6378849719	0.335056032231
48	Tupaia belangeri	25	24.3837586502	-38.8266371783	1.05518776781
49	Tursiops truncatus	27	23.9442746926	-40.0516794233	1.96505311345
50	Vicugna pacos	26	24.6166826812	-39.9796275855	1.40056030051
51	Xenopus tropicalis	26	25.3023816966	-31.7391849272	-4.29862164922
**Ensembl fungi**					
52	Aspergillus clavatus	16	23.8641230844	-34.7521931111	-0.320469244016
53	Aspergillus flavus	16	24.4225232971	-31.1402197478	-3.67368429775
54	Aspergillus fumigatus	16	23.9702886198	-34.1271770178	-0.94401115876
55	Aspergillus nidulans	15	23.9961385438	-34.012094714	-1.64567300129
56	Aspergillus niger	16	24.0051389055	-32.3251790574	-3.00194506058
57	Aspergillus oryzae	16	24.5685197236	-30.3977940129	-4.05280670219
58	Aspergillus terreus	16	23.9930725285	-32.5761534583	-2.15079615461
59	Neosartorya fischeri	15	23.9607788272	-34.0869333039	-1.13489174964
60	Neurospora crassa	16	22.1835811737	-45.4575826101	5.05946474512
61	Saccharomyces cerevisiae	13	26.2437745288	-39.6813468432	-0.868582921567
62	Schizosaccharomyces pombe	12	26.6846065899	-31.3851019399	-5.16557277407
**Ensembl bacteria**					
63	Bacillus amyloliquefaciens	9	27.85501654	-15.0819242125	-9.81758115734
64	Bacillus anthracis A0248	9	29.4986580025	-11.4001553892	-14.6463412911

Continued on next page

Table S2 – Ensembl species used in proteome analysis (continued)

#	Species	Node #	FILV	hydrophobicity	hydrophilicity
65	Bacillus anthracis Ames	9	29.5699411563	-10.5736637175	-15.1003651261
66	Bacillus anthracis Ames ancestor	9	29.5210990185	-11.4924203223	-14.6281520554
67	Bacillus anthracis CDC 684	9	29.4724616323	-11.940155966	-14.4445358971
68	Bacillus anthracis Sterne	9	29.5622919136	-10.6319323635	-15.126900634
69	Bacillus cereus 03BB102	8	29.4681897826	-11.8920449463	-14.4474911733
70	Bacillus cereus AH187	8	29.3671142406	-12.6276027932	-14.075567435
71	Bacillus cereus AH820	8	29.4279839747	-12.2283765882	-14.3222060772
72	Bacillus cereus ATCC 10987	8	29.4501123612	-12.2300770243	-14.4033175099
73	Bacillus cereus ATCC 14579	8	29.4117162356	-12.2698292478	-14.3385627207
74	Bacillus cereus B4264	8	29.3393123828	-12.6867195196	-14.0871953545
75	Bacillus cereus G9842	8	29.3223866005	-13.7450882327	-13.6068738981
76	Bacillus cereus Q1	8	29.4113990844	-12.2399194986	-14.3690907152
77	Bacillus cereus ZK	8	29.5406179507	-11.6944822078	-14.7284604812
78	Bacillus cereus cytotoxis	8	29.3266684199	-12.9183134485	-13.0710242952
79	Bacillus clausii	8	28.4495139601	-8.8787744915	-14.0160116852
80	Bacillus halodurans	8	28.8176917463	-14.1254678688	-12.1954859278
81	Bacillus licheniformis Goettingen	9	28.2007361755	-14.981358507	-10.0075138299
82	Bacillus licheniformis Novozymes	9	28.1951264893	-15.0456185584	-10.0041365699
83	Bacillus pumilus	8	28.5640317247	-15.1077055649	-9.52951248371
84	Bacillus subtilis	8	28.2801556673	-15.0337273743	-10.4496306475
85	Bacillus thuringiensis	9	29.374159023	-12.0470844418	-14.2602243975
86	Bacillus thuringiensis konkukian	9	29.5692165726	-10.9044837442	-15.0148516891
87	Bacillus weihenstephanensis	9	29.3154907109	-13.2166579267	-13.71164024
88	Borrelia afzelii	9	32.3179574012	-18.309401867	-10.6298180762
89	Borrelia burgdorferi DSM 4680	8	31.6049439813	-22.4622553157	-8.65571344333
90	Borrelia burgdorferi ZS7	8	31.778807605	-21.1786146257	-9.15901770789
91	Borrelia duttonii	8	31.4964132131	-19.4612446246	-7.95947977319
92	Borrelia garinii	9	32.0694659253	-18.9486079026	-9.97827435504
93	Borrelia hermsii	8	32.5175714619	-12.2651417739	-12.7888532291
94	Borrelia recurrentis	8	32.4119934445	-15.2231233029	-11.1606403116
95	Borrelia turicatae	8	32.691436181	-11.9855468559	-13.2291586778
96	Buchnera aphidicola 5A	8	31.355978409	-15.5151845592	-10.7063409847
97	Buchnera aphidicola Baizongia	8	32.1458761743	-7.92588684584	-15.1493114467
98	Buchnera aphidicola Cinara	8	32.6307598337	-17.6399220169	-8.52384259453
99	Buchnera aphidicola Schizaphis	8	31.6492264944	-15.7515629996	-9.93373102072
100	Buchnera aphidicola Tokyo 1998	8	31.3512739158	-15.3080531002	-10.7281640969
101	Buchnera aphidicola Tuc7	8	31.3797735559	-15.4310387367	-10.7227019766
102	Escherichia coli 55989	8	27.3364411676	-9.42039509602	-11.6332958768
103	Escherichia coli ATCC 27325	8	27.6466628226	-6.83041711745	-12.6950055283
104	Escherichia coli ATCC 8739	8	27.6014450941	-7.13915462728	-12.3915646489
105	Escherichia coli BL21	8	27.6380804675	-6.37178923411	-12.8254582193
106	Escherichia coli BL21 DE3 JGI	8	27.673043372	-6.20967920502	-12.8968134289
107	Escherichia coli BL21 DE3 KRIBB	8	27.6385446614	-6.51833662521	-12.8419443152
108	Escherichia coli BW2952	8	27.6769451151	-6.54268571287	-12.7195721704
109	Escherichia coli DH10B	8	27.6821301312	-6.44435141799	-12.8515051013
110	Escherichia coli EC4115	8	27.1001214542	-11.2126221115	-10.6287867317
111	Escherichia coli EDL933	8	27.0935574607	-11.1557148498	-10.8631392726
112	Escherichia coli K12	8	27.5884491667	-7.62283105405	-11.8382248007
113	Escherichia coli O103 H2 12009	8	27.1115975725	-10.7952068166	-10.728311188
114	Escherichia coli O111 H 11128	8	27.1776551649	-10.3005574771	-10.8966649133
115	Escherichia coli O127 H6	8	27.3845322443	-8.8396188704	-11.4144652082

Continued on next page

Table S2 – Ensembl species used in proteome analysis (continued)

#	Species	Node #	FILV	hydrophobicity	hydrophilicity
116	Escherichia coli O139 H28	8	27.4258575666	-9.28644107577	-11.6502092512
117	Escherichia coli O17 K52 H18	8	27.3856098302	-8.68901937569	-11.8634773844
118	Escherichia coli O1 K1 APEC	8	27.3125690175	-9.32485107286	-11.5860073464
119	Escherichia coli O26 H11 11368	8	26.9807996414	-11.411979679	-10.3923248372
120	Escherichia coli O45 K1	8	27.3841411685	-8.4009408743	-11.8360932357
121	Escherichia coli O6	8	27.5142546966	-8.07496808412	-12.4112547635
122	Escherichia coli O6 K15 H31	8	27.6258106238	-6.98959320665	-12.7731280192
123	Escherichia coli O7 K1	8	27.3883613488	-8.70979207634	-12.0049408408
124	Escherichia coli O8	8	27.5599959177	-7.18651221788	-12.5637575086
125	Escherichia coli O81	8	27.224900381	-9.55850076474	-11.2917364639
126	Escherichia coli O9 H4	8	27.6009144394	-7.08140921912	-12.3692307462
127	Escherichia coli REL606	8	27.606253013	-6.82563852271	-12.7780714204
128	Escherichia coli SE11	8	27.3071173135	-9.19007382723	-11.6731120053
129	Escherichia coli SMS 3 5	8	27.5670896155	-7.58314694573	-12.4408596888
130	Escherichia coli Sakai	8	27.020919607	-11.5316391864	-10.6060115218
131	Escherichia coli TW14359	8	27.0817161511	-11.1632953174	-10.6740842813
132	Escherichia coli UTI89	8	27.4559989969	-8.08044124787	-12.1602120774
133	Escherichia fergusonii	8	27.5917915973	-7.21844921554	-12.2541210218
134	Mycobacterium abscessus	11	25.9996898455	-2.1177680874	-10.7845185681
135	Mycobacterium avium	10	25.3860476594	-4.13531834632	-8.69121085503
136	Mycobacterium bovis AF2122 97	11	25.4587503269	-0.319362422318	-10.6557793287
137	Mycobacterium bovis Pasteur 1173P2	11	25.4491090543	-0.507553455985	-10.4928268303
138	Mycobacterium bovis Tokyo 172	11	25.4674245923	-0.217655494252	-10.6854978368
139	Mycobacterium gilvum	10	25.6142525632	-3.22181035605	-9.34828560706
140	Mycobacterium leprae Br4923	10	26.9490732316	0.733300157092	-11.0716606813
141	Mycobacterium leprae TN	10	26.9424761594	0.695383724319	-11.0594581951
142	Mycobacterium marinum	10	25.1005651725	-0.410481563512	-11.8273927736
143	Mycobacterium paratuberculosis	12	25.3213637926	-4.47713683867	-8.14385908792
144	Mycobacterium smegmatis	10	26.0278706169	-1.26218395362	-10.614461187
145	Mycobacterium sp JLS	12	25.7399387866	-2.72064590493	-9.57108846287
146	Mycobacterium sp KMS	12	25.5716454094	-3.7759167687	-9.0250897997
147	Mycobacterium sp MCS	12	25.6007066461	-3.88815705582	-8.90484084318
148	Mycobacterium tuberculosis ATCC 25177	10	25.4762553312	-0.386250592357	-10.7448740098
149	Mycobacterium tuberculosis CDC1551	10	25.3366309426	-1.50767264771	-10.0219164876
150	Mycobacterium tuberculosis H37Rv	10	25.4344169459	-0.449983568773	-10.7581345804
151	Mycobacterium tuberculosis KZN 1435	10	25.3983011673	-0.66999753609	-10.6751412282
152	Mycobacterium ulcerans	10	25.509442272	-1.2917212745	-10.4748590851
153	Mycobacterium vanbaalenii	10	25.7406410354	-1.9485933759	-9.94938047557
154	Neisseria gonorrhoeae ATCC 700825	8	26.4666886162	-15.9843921017	-6.6731328807
155	Neisseria gonorrhoeae NCCP11945	8	26.1615127142	-17.8691549683	-6.07764650047
156	Neisseria meningitidis 2a	8	26.7124669908	-15.1713200049	-7.57035202315
157	Neisseria meningitidis A	8	26.7469828899	-14.5504882344	-7.62974361129
158	Neisseria meningitidis B	8	26.5827362823	-15.8470198797	-7.01355064371
159	Neisseria meningitidis C	8	26.6792184179	-15.8759645015	-7.24744980754
160	Neisseria meningitidis alpha14	8	26.7144884824	-14.1643706155	-7.86624458407
161	Pyrococcus abyssi	8	31.1843179457	-7.08909752115	-15.6560969274
162	Pyrococcus furiosus	8	31.1161186511	-7.66337960714	-16.8295552822
163	Pyrococcus horikoshii	8	31.2987471853	-4.49751177315	-18.1293034195
164	Pyrococcus kodakaraensis	8	30.0555655697	-9.4829502261	-16.0938893548
165	Shigella boydii 18	8	27.2142530954	-11.3714976956	-10.2537075602
166	Shigella boydii 4	8	27.1342134435	-11.7127400988	-10.4534838255

Continued on next page

Table S2 – Ensembl species used in proteome analysis (continued)

#	Species	Node #	FILV	hydrophobicity	hydrophilicity
167	Shigella dysenteriae	8	27.1307699427	-11.8266043757	-10.4986049652
168	Shigella flexneri 2457T	8	27.350651336	-9.39475185061	-11.2576340575
169	Shigella flexneri 301	8	27.2479921706	-10.7602954655	-10.9015503394
170	Shigella flexneri 5b	8	27.3027949223	-10.0203509812	-11.2748831447
171	Shigella sonnei	8	27.138563728	-12.0687190499	-10.3104876608
172	Staphylococcus aureus COL	8	28.9294768083	-19.9979904987	-8.13228444234
173	Staphylococcus aureus JH1	8	28.7975749823	-21.1840830034	-7.6111394961
174	Staphylococcus aureus JH9	8	28.7657199639	-21.3796305178	-7.48768351743
175	Staphylococcus aureus MRSA252	8	28.9606936827	-20.270798557	-8.0584039007
176	Staphylococcus aureus MSSA476	8	29.157092472	-18.6544847681	-8.93949891895
177	Staphylococcus aureus MW2	8	28.9721395348	-19.9738280094	-8.23631274767
178	Staphylococcus aureus Mu3	8	28.8625054366	-20.7819161332	-7.83475130197
179	Staphylococcus aureus Mu50	8	28.8391476942	-21.1169632588	-7.72261726624
180	Staphylococcus aureus N315	8	28.9346908684	-20.1846908684	-8.02533302827
181	Staphylococcus aureus NCTC 8325	8	29.0035903496	-19.6690819321	-8.48674525042
182	Staphylococcus aureus Newman	8	28.8570136801	-20.6767393774	-7.9290660408
183	Staphylococcus aureus TCH1516	8	28.9864439512	-20.0596559217	-8.2246320141
184	Staphylococcus aureus USA300	8	28.8347858419	-21.1750999254	-7.59586179522
185	Staphylococcus aureus bovine RF122	8	29.2776419721	-17.4810887345	-9.59254664462
186	Staphylococcus carnosus	8	28.5407270884	-20.7632802456	-8.11587945891
187	Staphylococcus epidermidis ATCC 12228	8	28.9830710958	-23.156899811	-7.32528935145
188	Staphylococcus epidermidis ATCC 35984	8	28.8372144502	-24.1301520719	-7.0507155348
189	Staphylococcus haemolyticus	8	28.7224171069	-23.16678801	-7.65482952886
190	Staphylococcus saprophyticus	8	29.3776243699	-15.4152821542	-11.0897335866
191	Streptococcus agalactiae III	8	29.0780659107	-16.6998698048	-8.6786661432
192	Streptococcus agalactiae Ia	8	29.0831201773	-15.8152950577	-9.18246536864
193	Streptococcus agalactiae V	8	29.1739192743	-15.6395310455	-9.22834402238
194	Streptococcus dysgalactiae	8	28.8210481396	-12.8654291201	-9.40922743611
195	Streptococcus equi	9	28.1563856885	-18.3003226828	-5.73669345792
196	Streptococcus equi MGCS10565	9	28.5517874332	-13.1874443192	-8.03343807753
197	Streptococcus equi zooepidemicus	9	28.5047932853	-14.5919266702	-7.63778152924
198	Streptococcus gordonii	8	28.78322989	-16.1949372724	-9.06158494725
199	Streptococcus mutans ATCC 700610	8	29.222736078	-14.7393279088	-9.12366981593
200	Streptococcus mutans NN2025	8	29.1993985864	-14.563478582	-8.98754488389
201	Streptococcus pneumoniae 19F	8	29.1338649059	-15.2945709117	-10.2178278588
202	Streptococcus pneumoniae 2	8	29.1482561203	-14.5111692599	-10.5450985213
203	Streptococcus pneumoniae 70585	8	28.9877835951	-15.3131575084	-10.1257849326
204	Streptococcus pneumoniae ATCC 700669	8	28.9424572572	-15.6840898348	-9.93357747686
205	Streptococcus pneumoniae ATCC BAA 255	8	29.1319221541	-14.9510525948	-10.3670246551
206	Streptococcus pneumoniae CGSP14	8	28.911957785	-16.1371674467	-9.8723654867
207	Streptococcus pneumoniae Hungary19A 6	8	28.7770075279	-16.2049861047	-9.73195195497
208	Streptococcus pneumoniae JJA	8	29.0611822349	-15.8073468589	-9.7979588499
209	Streptococcus pneumoniae P1031	8	29.0296853473	-15.7920057505	-9.82007376411
210	Streptococcus pneumoniae TIGR4	8	28.9770706691	-14.6197167651	-10.6066517992
211	Streptococcus pneumoniae Taiwan19F 14	8	29.043539862	-15.590234911	-9.96774315574
212	Streptococcus pyogenes ATCC BAA 595	8	28.5611510791	-15.4267662793	-7.54964028777
213	Streptococcus pyogenes M18	8	28.6063556003	-15.2404423557	-7.56023647011
214	Streptococcus pyogenes M2	8	28.6230622387	-15.4824291191	-7.91495904384
215	Streptococcus pyogenes M28	8	28.6762559477	-15.2023673994	-7.80422614356
216	Streptococcus pyogenes M4	8	28.6292537504	-15.855194273	-7.76768651795
217	Streptococcus pyogenes M49	8	28.7203476732	-14.0917818253	-8.23215977343

Continued on next page

Table S2 – Ensembl species used in proteome analysis (continued)

#	Species	Node #	FILV	hydrophobicity	hydrophilicity
218	Streptococcus pyogenes M5	8	28.7053738756	-13.9786942466	-8.17755184908
219	Streptococcus pyogenes M6	8	28.5637117602	-15.0463424393	-7.83416390819
220	Streptococcus pyogenes MGAS2096	8	28.6385550652	-15.0984239567	-8.01238081474
221	Streptococcus pyogenes MGAS5005	8	28.7599483414	-13.8767757636	-8.45209574611
222	Streptococcus pyogenes MGAS9429	8	28.6891690743	-14.8517758325	-7.99123709704
223	Streptococcus pyogenes SF370	8	28.6920272995	-14.3840739879	-8.00622382503
224	Streptococcus pyogenes SSI 1	8	28.5991325655	-14.9491313488	-7.76825608973
225	Streptococcus sanguinis	8	28.5025053138	-16.18229654	-8.62194254462
226	Streptococcus suis 05ZYH33	8	28.8876003656	-13.8642595304	-10.6824041224
227	Streptococcus suis 98HAH33	8	28.9039681514	-13.7619632785	-10.7222555842
228	Streptococcus suis BM407	8	28.996449367	-13.5965063362	-10.6313966044
229	Streptococcus suis P1 7	8	29.042135322	-12.1384438734	-11.2804982371
230	Streptococcus suis SC84	8	29.0531469502	-12.7263354456	-11.030891358
231	Streptococcus thermophilus ATCC BAA 250	8	29.1033668096	-14.1253261448	-9.61390512702
232	Streptococcus thermophilus ATCC BAA 491	8	29.0077539258	-15.2604575005	-8.85208687592
233	Streptococcus thermophilus CNRZ 1066	8	29.1330046044	-13.7285917992	-9.86273933536
234	Streptococcus uberis	8	29.3281000904	-12.5494159216	-10.3750407876
235	Wolbachia pipientis Culex pipiens	9	29.3157168798	-20.7267779354	-6.78180537226
236	Wolbachia pipientis wMel	9	29.4057565118	-17.8689893242	-7.944602012
237	Wolbachia sp Brugia malayi	9	30.0708576941	-12.5685914122	-10.1222544723
238	Wolbachia sp Drosophila simulans	9	29.2841799648	-19.4198927582	-7.20812509039
**Ensembl plants**					
239	Arabidopsis lyrata	18	25.9907926953	-30.4142522607	-3.2884463922
240	Arabidopsis thaliana	18	25.9178308516	-31.7059868556	-2.97277754913
241	Brachypodium distachyon	19	24.4235321738	-26.775790694	-2.18583027399
242	Oryza indica	19	24.0456993665	-26.5602535336	-2.00789982733
243	Oryza sativa	19	23.6643839517	-35.5393019989	1.43353694282
244	Populus trichocarpa	19	26.1394154193	-26.0388987149	-5.24728272092
245	Sorghum bicolor	19	24.2594019763	-27.1851305205	-2.13916519236
246	Vitis vinifera	18	26.8442144126	-22.0186390724	-8.49252677916
**Ensembl metazoa**					
247	Aedes aegypti	29	24.5280294153	-40.7636544656	1.50490256636
248	Anopheles gambiae	29	24.1510817843	-36.7226307329	-0.013704142103
249	Caenorhabditis brenneri	15	25.0582856857	-40.3114852103	0.199652933964
250	Caenorhabditis briggsae	15	25.3067565977	-37.3168267839	-1.33862650986
251	Caenorhabditis elegans	15	25.3194997509	-35.5108639787	-1.5426787175
252	Caenorhabditis japonica	15	25.4299465184	-33.8231826163	-1.40736059401
253	Caenorhabditis remanei	15	25.9102670775	-35.8447632606	-2.5612766037
254	Culex quinquefasciatus	29	24.3522910512	-39.5859710914	1.93985037954
255	Drosophila ananassae	37	23.4053095693	-43.5362820753	3.82133629176
256	Drosophila erecta	36	23.4657460297	-42.5538112401	3.73887568958
257	Drosophila grimshawi	38	23.337972338	-42.9033783942	4.18711643623
258	Drosophila melanogaster	36	23.159014235	-44.93002556	4.92973342304
259	Drosophila mojavensis	37	23.2723307924	-43.237610704	4.13540973514
260	Drosophila persimilis	36	23.3198166961	-42.1465045041	3.5079096259
261	Drosophila pseudoobscura	36	23.1283195518	-43.1236422382	3.94878907959
262	Drosophila sechellia	36	23.5496499297	-42.3297911341	3.47842458723
263	Drosophila simulans	36	23.7352164513	-41.0545267776	2.62127455847
264	Drosophila virilis	35	23.3085895285	-42.2995975802	3.80200595997
265	Drosophila willistoni	36	23.3910162696	-44.2478190693	3.88596914514
266	Drosophila yakuba	36	23.4816851308	-42.7046155019	3.56122272399

Continued on next page

Table S2 – Ensembl species used in proteome analysis (continued)

#	Species	Node #	FILV	hydrophobicity	hydrophilicity
267	Ixodes scapularis	20	24.8213847617	-28.826774208	-2.26774173639
268	Pediculus humanus	23	25.3323742457	-47.9811410337	2.7897692826

S6 TREES

S6.1 Node number is dependent on the "universal" tree of life (not the pruned tree).

Thre pruned tree of life was obtained from iToL with the option to leave "Internal nodes expanded", which means that the node number obtained for each species is dependent not on the number of sequenced species in the tree of life, but on the number of species in the NCBI taxonomy database. For simplicity, each branch length is set to 1 (and hense , node number is the metric for time). Below is an example of two trees in newick format obtained using the "Internal nodes expanded", which explains how, even with increasing the number of species in the tree of life, the node number of *Homo sapiens*, and *Mus musculus* do not change. Given that the number of sequenced species (hundreds) is much less than the number of species in the NCBI taxonomy database (as of 2012, NCBI records $249,450$ species), we can conclude that, barring the refreshing of the NCBI taxonomy database, the addition of more sequenced species to our ToL will not change the node numbers of the extant species.

LESS COMPREHENSIVE TREE
SPECIES USED: Homo sapiens,Mus musculus.
NODE NUMBERS OBTAINED FROM TREE: Homo sapiens:30; Mus musculus:30.
PRUNED TREE IN NEWICK FORMAT: (Homo sapiens) Homo) Homininae) Hominidae) Hominoidea) Catarrhini) Simiiformes) Haplorrhini) Primates,((((((((Mus musculus) Mus) Mus) Murinae) Muridae) Muroidea) Sciurognathi) Rodentia) Glires) Euarchontoglires) Eutheria) Theria) Mammalia) Amniota) Tetrapoda) Sarcopterygii) Euteleostomi) Teleostomi) Gnathostomata) Vertebrata) Craniata) Chordata) Deuterostomia) Coelomata) Bilateria) Eumetazoa) Metazoa) Opisthokonta) Eukaryota) cellular organisms) ;

MORE COMPREHENSIVE TREE
SPECIES USED: Homo sapiens, Mus musculus, Escherichia coli.
NODE NUMBERS OBTAINED FROM TREE: Escherichia coli:8; Homo sapiens: 30; Mus musculus:30;
PRUNED TREE IN NEWICK FORMAT: (Homo sapiens) Homo) Homininae) Hominidae) Hominoidea) Catarrhini) Simiiformes) Haplorrhini) Primates,((((((((Mus musculus) Mus) Mus) Murinae) Muridae) Muroidea) Sciurognathi) Rodentia) Glires) Euarchontoglires) Eutheria) Theria) Mammalia) Amniota) Tetrapoda) Sarcopterygii) Euteleostomi) Teleostomi) Gnathostomata) Vertebrata) Craniata) Chordata) Deuterostomia) Coelomata) Bilateria) Eumetazoa) Metazoa) Opisthokonta) Eukaryota,((((((Escherichia coli) Escherichia) Enterobacteriaceae) Enterobacteriales) Gammaproteobacteria) Proteobacteria) Bacteria) cellular organisms) ;

S6.2 THE COMMON TREE USED TO CALCULATE NODE SPACE

The following “tree” in newick format was obtained from iTol’s NCBI algorithm. Each bracket pair indicates an additional node that separates the species (or group of species) from the last common ancestor. The node number of a species in Fig. 2 onwards is the number of brackets that the species is embedded into.

((((((((Thermococcus kodakarensis) Thermococcus, (Pyrococcus horikoshii, Pyrococcus furiosus, Pyrococcus abyssi) Pyrococcus) Thermococcaceae) Thermococcales) Thermococci) Euryarchaeota) Archaea, ((((((Borrelia duttonii, (Borrelia afzelii, Borrelia garinii, Borrelia burgdorferi) Borrelia burgdorferi group, Borrelia turicatae, Borrelia hermsii, Borrelia recurrentis) Borrelia) Spirochaetaceae) Spirochaetales) Spirochaetes —class—) Spirochaetes, (((((Bacillus clausii, Bacillus pumilus, (Bacillus licheniformis, Bacillus subtilis, Bacillus amyloliquefaciens) Bacillus subtilis group, (Bacillus weihenstephanensis, Bacillus anthracis, Bacillus thuringiensis, Bacillus cereus) Bacillus cereus group, Bacillus halodurans) Bacillus) Bacillaceae, ((Staphylococcus saprophyticus, Staphylococcus epidermidis, Staphylococcus carnosus, Staphylococcus haemolyticus, Staphylococcus aureus) Staphylococcus) Staphylococcaceae) Bacillales, (((Streptococcus thermophilus, Streptococcus pyogenes, Streptococcus gordonii, Streptococcus sanguinis, Streptococcus uberis, Streptococcus pneumoniae, Streptococcus agalactiae, Streptococcus suis, Streptococcus mutans, (Streptococcus equi, Streptococcus dysgalactiae) Streptococcus dysgalactiae group) Streptococcus) Streptococcaceae) Lactobacillales) Bacilli) Firmicutes, ((((((Wolbachia pipientis) Wolbachia) Wolbachieae) Rickettsiaceae) Rickettsiales) Alphaproteobacteria, ((((Buchnera aphidicola) Buchnera, (Shigella boydii, Shigella flexneri, Shigella sonnei, Shigella dysenteriae) Shigella, (Escherichia fergusonii, Escherichia coli) Escherichia) Enterobacteriaceae) Enterobacteriales) Gammaproteobacteria, ((((Neisseria meningitidis, Neisseria gonorrhoeae) Neisseria) Neisseriaceae) Neisseriales) Betaproteobacteria) Proteobacteria, (((((((Mycobacterium marinum, ((Mycobacterium avium subsp. paratuberculosis) Mycobacterium avium) Mycobacterium avium complex —MAC—, Mycobacterium leprae, Mycobacterium vanbaalenii, Mycobacterium smegmatis, Mycobacterium gilvum, ((Mycobacterium abscessus) Mycobacterium abscessus subgroup) Mycobacterium chelonae group, (Mycobacterium tuberculosis, Mycobacterium bovis) Mycobacterium tuberculosis complex, Mycobacterium ulcerans) Mycobacterium) Mycobacteriaceae) Corynebacterineae) Actinomycetales) Actinobacteridae) Actinobacteria —class—) Actinobacteria) Bacteria, (((((Dictyostelium discoideum) Dictyostelium) Dictyosteliida) Mycetozoa) Amoebozoa, ((((((((((((((((Sorghum bicolor) Sorghum) Andropogoneae) Panicoideae) PACCAD clade, ((((Oryza sativa) Oryza) Oryzeae) Ehrhartoideae, (((Brachypodium distachyon) Brachypodium) Brachypodieae) Pooideae) BEP clade) Poaceae) Poales) commelinids) Liliopsida, (((((((Vitis vinifera) Vitis) Vitaceae) Vitales) rosids incertae sedis, (((((Populus trichocarpa) Populus) Saliceae) Salicaceae) Malpighiales) fabids, ((((Arabidopsis lyrata, Arabidopsis thaliana) Arabidopsis) Brassicaceae) Brassicales) malvids) rosids) core eudicotyledons) eudicotyledons) Magnoliophyta) Spermatophyta) Euphyllophyta) Tracheophyta) Embryophyta) Streptophytina) Streptophyta) Viridiplantae, ((((((Plasmodium knowlesi, Plasmodium vivax) Plasmodium —Plasmodium—, (Plasmodium falciparum) Plasmodium —Laverania—) Plasmodium) Haemosporida) Aconoidasida) Apicomplexa) Alveolata, (((((((((((Caenorhabditis elegans, Caenorhabditis remanei, Caenorhabditis japonica, Caenorhabditis briggsae, Caenorhabditis brenneri) Caenorhabditis) Peloderinae) Rhabditidae) Rhabditoidea) Rhabditida) Chromadorea) Nematoda) Pseudocoelomata, (((((((((Ciona savignyi, Ciona intestinalis) Ciona) Cionidae) Phlebobranchia) Enterogona) Ascidiacea) Tunicata, (Tetraodon nigroviridis) Tetraodon, (Takifugu rubripes) Takifugu) Tetraodontidae) Tetradontoidea) Tetraodontoidei) Tetraodontiformes, (((((Gasterosteus aculeatus) Gasterosteus) Gasterosteidae) Gasterosteiformes) Gasterosteiformes/Syngnathiformes group, ((((((Oryzias latipes) Oryzias) Oryziinae) Adrianichthyidae) Adrianichthyoidei) Beloniformes) Atherinomorpha) Smegmamorpha) Percomorpha) Euacanthopterygii) Acanthopterygii) Holacanthopterygii) Euacanthomorpha) Acanthomorpha) Ctenosquamata) Eurypterygii) Neoteleostei) Neognathi) Euteleostei, ((((((((Danio rerio) Danio) Cyprinidae) Cyprinoidea) Cypriniformes) Cypriniphysi) Otophysi) Ostariophysi) Otocephala) Clupeocephala) Elopocephala) Teleostei) Neopterygii) Actinopteri) Actinopterygii, (((((((((((Xenopus —Silurana— tropicalis) Silurana) Xenopus) Xenopodinae) Pipidae) Pipoidea) Mesobatrachia) Anura) Batrachia) Amphibia, ((((((((((Tarsius syrichta) Tarsius) Tarsiidae) Tarsiiformes, ((((((Homo sapiens) Homo, (Gorilla gorilla) Gorilla, (Pan troglodytes) Pan) Homininae, ((Pongo pygmaeus) Pongo) Ponginae) Hominidae) Hominoidea, ((((Macaca mulatta) Macaca) Cercopithecinae) Cercopithecidae) Cercopithecoidea) Catarrhini, ((((Callithrix jacchus) Callithrix) Callitrichinae) Cebidae) Platyrrhini) Simiiformes) Haplorrhini, ((((Microcebus murinus) Microcebus) Cheirogaleidae) Lemuriformes, (((Otolemur garnettii) Otolemur) Galagidae) Lorisiformes) Strepsirrhini) Primates, ((((Oryctolagus cuniculus) Oryctolagus) Leporidae, ((Ochotona princeps) Ochotona) Ochotonidae) Lagomorpha, ((((((Spermophilus tridecemlineatus) Spermophilus) Marmotini) Xerinae) Sciuridae, ((((Mus musculus) Mus, (Rattus norvegicus) Rattus) Murinae) Muridae) Muroidea, (((Dipodomys ordii) Dipodomys) Dipodomyinae) Heteromyidae) Sciurognathi, (((Cavia porcellus) Cavia) Caviidae) Hystricognathi) Rodentia) Glires, (((Tupaia belangeri) Tupaia) Tupaiidae) Scandentia) Euarchontoglires, (((Dasypus novemcinctus) Dasypus) Dasypodidae, ((Choloepus hoffmanni) Choloepus) Megalonychidae) Xenarthra, (((((Equus caballus) Equus subg. Equus) Equus) Equidae) Perissodactyla, ((((Erinaceus europaeus) Erinaceus) Erinaceinae) Erinaceidae, (((Sorex araneus) Sorex) Soricinae) Soricidae) Insectivora, ((((((Bos taurus) Bos) Bovinae) Bovidae) Pecora) Ruminantia, (((Lama pacos) Lama) Camelidae) Tylopoda, ((((Tursiops truncatus) Tursiops) Delphinidae) Odontoceti) Cetacea, (((Sus scrofa) Sus) Suidae) Suina) Cetartiodactyla, (((((Pteropus vampyrus) Pteropus) Pteropodinae) Pteropodidae) Megachiroptera, (((Myotis lucifugus) Myotis) Vespertilionidae) Microchiroptera) Chiroptera, (((((Felis catus) Felis) Felinae) Felidae) Feliformia, ((((Canis lupus familiaris) Canis lupus) Canis) Canidae) Caniformia) Carnivora) Laurasiatheria, ((((Echinops telfairi) Echinops) Tenrecinae) Tenrecidae, (((Procavia capensis) Procavia) Procaviidae) Hyracoidea, (((Loxodonta africana) Loxodonta) Elephantidae) Proboscidea) Afrotheria) Eutheria, ((((Macropus eugenii) Macropus) Macropodidae) Diprotodontia, ((((Monodelphis domestica) Monodelphis) Didelphinae) Didelphidae) Didelphimorphia) Metatheria) Theria, ((((Ornithorhynchus anatinus) Ornithorhynchus) Ornithorhynchidae) Monotremata) Prototheria) Mammalia, (((((((((((((Taeniopygia guttata) Taeniopygia) Estrildinae) Estrildidae) Passeroidea) Passeriformes, ((((Gallus gallus) Gallus) Phasianinae, ((Meleagris gallopavo) Meleagris) Meleagridinae) Phasianidae) Galliformes) Neognathae) Aves) Coelurosauria) Theropoda) Saurischia) Dinosauria) Archosauria, ((((((Anolis carolinensis) Anolis) Polychrotinae) Iguanidae) Iguania) Squamata) Lepidosauria) Sauria) Sauropsida) Amniota) Tetrapoda) Sarcopterygii) Euteleostomi) Teleostomi) Gnathostomata) Vertebrata) Craniata) Chordata) Deuterostomia, (Drosophila pseudoobscura, Drosophila persimilis) pseudoobscura subgroup) obscura group, ((Drosophila willistoni) willistoni subgroup) willistoni group, (((Drosophila ananassae) ananassae species complex) ananassae subgroup, (Drosophila erecta, Drosophila yakuba, Drosophila sechellia, Drosophila melanogaster, Drosophila simulans) melanogaster subgroup) melanogaster group) Sophophora, (((((Drosophila grimshawi) grimshawi subgroup) grimshawi group) grimshawi clade) picture wing clade) Hawaiian Drosophila, ((((Drosophila mojavensis) mojavensis species complex) mulleri subgroup) repleta group, (Drosophila virilis) virilis group) Drosophila) Drosophila) Drosophiliti) Drosophilina) Drosophilini) Drosophilinae) Drosophilidae) Ephydroidea) Acalyptratae) Schizophora) Cyclorrhapha) Eremoneura) Muscomorpha) Brachycera, (((((((((Anopheles gambiae) gambiae species complex) Pyretophorus) Cellia) Anopheles) Anophelinae, (((((Aedes aegypti) Stegomyia) Aedes) Aedes/Ochlerotatus group, (((Culex quinquefasciatus) Culex pipiens complex) Culex) Culex) Culicini) Culicinae) Culicidae) Culicoidea) Culicimorpha) Nematocera) Diptera) Endopterygota, (((((Pediculus humanus) Pediculus) Pediculidae) Anoplura) Phthiraptera) Paraneoptera) Neoptera) Pterygota) Dicondylia) Insecta) Hexapoda) Pancrustacea) Mandibulata, (((((((((Ixodes scapularis) Ixodes) Ixodinae) Ixodidae) Ixodoidea) Ixodida) Parasitiformes) Acari) Arachnida) Chelicerata) Arthropoda) Panarthropoda) Protostomia) Coelomata) Bilateria) Eumetazoa) Metazoa, ((((((((Schizosaccharomyces pombe) Schizosaccharomyces) Schizosaccharomycetaceae) Schizosaccharomycetales) Schizosaccharomycetes) Taphrinomycotina, ((((((Saccharomyces cerevisiae) Saccharomyces) Saccharomycetaceae) Saccharomycetales) Saccharomycetes) Saccharomycotina, ((((((((Neurospora crassa) Neurospora) Sordariaceae) Sordariales) Sordariomycetidae) Sordariomycetes) Sordariomyceta, ((((((Aspergillus terreus, Aspergillus niger, Aspergillus clavatus, Aspergillus oryzae, Aspergillus flavus) Aspergillus) mitosporic Trichocomaceae, (Emericella nidulans) Emericella, ((Neosartorya fischeri) Neosartorya fischeri group, (Aspergillus fumigatus) Neosartorya fumigata) Neosartorya) Trichocomaceae) Eurotiales) Eurotiomycetidae) Eurotiomycetes) Leotiomyceta) Pezizomycotina) Saccharomyceta) Ascomycota) Dikarya) Fungi) Fungi/Metazoa group) Eukaryota) cellular organisms);

References and Notes

1. Hartl, D. L. & Clark, A. G. *Principles of population genetics*, chap. Molecular population genetics, 317–386 (Sinauer Associates, Inc. Publishers, 2006), 4th edn.

2. Mindell, D., Sites, J. & Graur, D. Mode of allozyme evolution: Increased genetic distance associated with speciation events. *J. evol. Biol* **3**, 125–131 (1990).

3. Barraclough, T. G. & Savolainen, V. Evolutionary rates and species diversity in flowering plants. *Evolution* **55**, 677–683 (2001).

4. Lanfear, R., Ho, S. Y. W., Love, D. & Bromham, L. Mutation rate is linked to diversification in birds. *Proc Natl Acad Sci U S A* **107**, 20423–20428 (2010).

5. Lanfear, R., Bromham, L. & Ho, S. Y. W. Reply to englund: Molecular evolution and diversification–counting species is better than counting nodes when the phylogeny is unknown. *PNAS* **108**, E85–E86 (2011).

6. Eo, S. H. & DeWoody, J. A. Evolutionary rates of mitochondrial genomes correspond to diversification rates and to contemporary species richness in birds and reptiles. *Proc Biol Sci* **277**, 3587–3592 (2010).

7. Webster, A. J., Payne, R. J. H. & Pagel, M. Molecular phylogenies link rates of evolution and speciation. *Science* **301**, 478 (2003).

8. Webster, A. J., Payne, R. J. H. & Pagel, M. Response to comments on "molecular phylogenies link rates of evolution and speciation". *Science* **303**, 173c (2004).

9. Venditti, C., Meade, A. & Pagel, M. Detecting the node-density artifact in phylogeny reconstruction. *Syst Biol* **55**, 637–643 (2006).

10. Venditti, C. & Pagel, M. Model misspecification not the node-density artifact. *Evolution* **62**, 2125–2126 (2008).

11. Venditti, C. & Pagel, M. Speciation as an active force in promoting genetic evolution. *Trends Ecol Evol* **25**, 14–20 (2010).

12. Pagel, M., Venditti, C. & Meade, A. Large punctuational contribution of speciation to evolutionary divergence at the molecular level. *Science* **314**, 119–121 (2006).

13. Smith, J. M. & Haigh, J. The hitch-hiking effect of a favourable gene. *Genet Res* **23**, 23–35 (1974).

14. Barton, N. H. Genetic hitchhiking. *Philos Trans R Soc Lond B Biol Sci* **355**, 1553–1562 (2000).

15. Smith, J. M. & Haigh, J. The hitch-hiking effect of a favourable gene. *Genet Res* **89**, 391–403 (2007).

16. Templeton, A. R. The reality and importance of founder speciation in evolution. *Bioessays* **30**, 470–479 (2008).

17. Henikoff, S. & Henikoff, J. G. Amino acid substitution matrices from protein blocks. *Proc Natl Acad Sci U S A* **89**, 10915–10919 (1992).

18. Arbiza, L., Patricio, M., Dopazo, H. & Posada, D. Genome-wide heterogeneity of nucleotide substitution model fit. *Genome Biol Evol* **3**, 896–908 (2011).

19. Abascal, F., Zardoya, R. & Posada, D. Prottest: selection of best-fit models of protein evolution. *Bioinformatics* **21**, 2104–2105 (2005).

20. Zuckerkandl, E. & Pauling, L. B. *Molecular disease, evolution, and genetic heterogeneity*, 189–225 (Academic Press, New York, 1962).

21. Zuckerkandl, E. & Pauling, L. Molecules as documents of evolutionary history. *J Theor Biol* **8**, 357–366 (1965).

22. Marigoliash, E. Primary structure and evolution of cytochrome c. *Proc Natl Acad Sci U S A* **50**, 672–679 (1963).

23. Kimura, M. Evolutionary rate at the molecular level. *Nature* **217**, 624–626 (1968).

24. King, J. L. & Jukes, T. H. Non-darwinian evolution. *Science* **164**, 788–798 (1969).

25. Kimura, M. Model of effectively neutral mutations in which selective constraint is incorporated. *Proc Natl Acad Sci U S A* **76**, 3440–3444 (1979).

26. Ohta, T. Slightly deleterious mutant substitutions in evolution. *Nature* **246**, 96–98 (1973).

27. Kimura, M. & Ota, T. On some principles governing molecular evolution. *Proc Natl Acad Sci U S A* **71**, 2848–2852 (1974).

28. Kimura, M. Possibility of extensive neutral evolution under stabilizing selection with special reference to nonrandom usage of synonymous codons. *Proc Natl Acad Sci U S A* **78**, 5773–5777 (1981).

29. Ohta & Gillespie. Development of neutral and nearly neutral theories. *Theor Popul Biol* **49**, 128–142 (1996).

30. Bromham, L. Molecular clocks and explosive radiations. *J Mol Evol* **57 Suppl 1**, S13–S20 (2003).

31. Gillespie, J. H. The molecular clock may be an episodic clock. *Proc Natl Acad Sci U S A* **81**, 8009–8013 (1984).

32. Gillespie, J. H. Natural selection and the molecular clock. *Mol Biol Evol* **3**, 138–155 (1986).

33. Murphy, R. W. & Lovejoy, N. R. Punctuated equilibrium or gradualism in the lizard genus sceloporus? lost in plesiograms and a forest of trees. *Cladistics* **14**, 95–103 (1998).

34. Fitch, W. M. & Bruschi, M. The evolution of prokaryotic ferredoxins–with a general method correcting for unobserved substitutions in less branched lineages. *Mol Biol Evol* **4**, 381–394 (1987).

35. Fitch, W. M. & Beintema, J. J. Correcting parsimonious trees for unseen nucleotide substitutions: the effect of dense branching as exemplified by ribonuclease. *Mol Biol Evol* **7**, 438–443 (1990).

36. Smith, S. A. & Donoghue, M. J. Rates of molecular evolution are linked to life history in flowering plants. *Science* **322**, 86–89 (2008).

37. Lanfear, R., Welch, J. J. & Bromham, L. Watching the clock: studying variation in rates of molecular evolution between species. *Trends Ecol Evol* **25**, 495–503 (2010).

38. Zeldovich, K. B., Berezovsky, I. N. & Shakhnovich, E. I. Protein and dna sequence determinants of thermophilic adaptation. *PLoS Comput Biol* **3**, e5 (2007).

39. Jordan, I. K. *et al.* A universal trend of amino acid gain and loss in protein evolution. *Nature* **433**, 633–638 (2005).

40. Tatusov, R. L., Koonin, E. V. & Lipman, D. J. A genomic perspective on protein families. *Science* **278**, 631–637 (1997).

41. Pagel, M. Inferring the historical patterns of biological evolution. *Nature* **401**, 877–884 (1999).

42. Freckleton, R. P., Harvey, P. H. & Pagel, M. Phylogenetic analysis and comparative data: a test and review of evidence. *Am Nat* **160**, 712–726 (2002).

43. Pagel, M. Inferring evolutionary processes from phylogenies. *Zoologica Scripta* **26**, 331–348 (1997).

44. Kersey, P. J. *et al.* Ensembl genomes: extending ensembl across the taxonomic space. *Nucleic Acids Res* **38**, D563–D569 (2010).
